# Predicting the dynamics of bacterial growth inhibition by ribosome-targeting antibiotics

**DOI:** 10.1088/1478-3975/aa8001

**Published:** 2017-11-16

**Authors:** Philip Greulich, Jakub Doležal, Matthew Scott, Martin R Evans, Rosalind J Allen

**Affiliations:** 1 Mathematical Sciences, University of Southampton, Highfield Campus, SO17 1BJ, United Kingdom; 2 Institute for Life Sciences, University of Southampton, Highfield Campus, SO17 1BJ, United Kingdom; 3 School of Physics and Astronomy, University of Edinburgh, Peter Guthrie Tait Road, Edinburgh EH9 3FD, United Kingdom; 4 Department of Applied Mathematics, University of Waterloo, Waterloo, ON, Canada; 5 Centre for Synthetic and Systems Biology, The University of Edinburgh, Edinburgh, United Kingdom

**Keywords:** antibiotics, bacterial growth, dynamical systems, ribosomes, pharmacodynamics

## Abstract

Understanding how antibiotics inhibit bacteria can help to reduce antibiotic use and hence avoid antimicrobial resistance—yet few theoretical models exist for bacterial growth inhibition by a clinically relevant antibiotic treatment regimen. In particular, in the clinic, antibiotic treatment is time-dependent. Here, we use a theoretical model, previously applied to steady-state bacterial growth, to predict the dynamical response of a bacterial cell to a time-dependent dose of ribosome-targeting antibiotic. Our results depend strongly on whether the antibiotic shows reversible transport and/or low-affinity ribosome binding (‘low-affinity antibiotic’) or, in contrast, irreversible transport and/or high affinity ribosome binding (‘high-affinity antibiotic’). For low-affinity antibiotics, our model predicts that growth inhibition depends on the duration of the antibiotic pulse, and can show a transient period of very fast growth following removal of the antibiotic. For high-affinity antibiotics, growth inhibition depends on peak dosage rather than dose duration, and the model predicts a pronounced post-antibiotic effect, due to hysteresis, in which growth can be suppressed for long times after the antibiotic dose has ended. These predictions are experimentally testable and may be of clinical significance.

## Introduction

Modern clinical practice relies on the use of antibiotics to combat bacterial infections, yet our knowledge of how antibiotics inhibit bacteria is surprisingly incomplete. In particular, mathematical models are needed to translate known information about the molecular processes that are targeted by antibiotics into predictions for bacterial growth rate as a function of antibiotic concentration. Such models would allow optimisation of dosing regimes [1–3], and provide a basis for understanding the evolution of resistance to antibiotics [4–7]. Recent work has focused on predicting how bacterial growth responds to a fixed antibiotic concentration [2, 7–10]. Although in the clinic the antibiotic concentration to which an infection is exposed is time-varying, there has been little mechanistic modelling of the response of bacterial growth to a time-varying dose of antibiotic (for recent work in this direction see [11–13]). In this paper, we present theoretical predictions for the dynamical changes in bacterial growth rate in response to a time-varying concentration of a ribosome-targeting antibiotic. Our analysis predicts qualitative, and potentially clinically relevant, differences in the dynamical response of bacterial growth to antibiotic treatment, depending on the molecular parameters for antibiotic-ribosome binding and transport of antibiotic across the bacterial cell boundary.

We focus here on antibiotics that target bacterial ribosomes. Ribosomes are multi-component, molecular machines which carry out protein synthesis—a function that is crucial for growth. Different ribosome-targeting antibiotics can bind to different components of the bacterial ribosome and inhibit different steps in protein synthesis [14]. In recent experimental and theoretical work [8], we showed that some ribosome-targeting antibiotics work better for bacteria that are growing rapidly (on a rich medium) while others work better for bacteria that are growing slowly (on a poor medium). These observations can be reproduced by a simple mathematical model that takes account of the molecular processes of antibiotic-ribosome binding and antibiotic transport across the cell boundary, as well as the physiological processes of cell growth and ribosome synthesis [8]. Reference [8], however, considered only the response to a fixed (time-invariant) antibiotic concentration. In the present paper, we extend the predictions of the model to the more clinically-relevant case of a time-dependent antibiotic dose.

Pharmacokinetic curves describe the time-varying local antibiotic concentration at an infection site during a clinical treatment regime [15]. These curves show a peak, since the antibiotic concentration initially increases following ingestion, then later decreases due to metabolism and excretion [15, 16]. Pharmacodynamics attempts to link these curves to the efficacy of antibiotic action [15, 16]. In particular, some dosing protocols are designed to maximise the peak concentration, whereas others aim to maximise the time at which the concentration is maintained above a certain threshold, or, alternatively, the area of the curve which is above a threshold [15]. Importantly, for some antibiotics, activity can persist for some time after the antibiotic is removed: this is known as the post-antibiotic effect [17, 18] and occurs for a variety of antibiotics including ribosome-targeting aminoglycosides [18, 19]. The mechanisms behind this effect are unknown but may include slow recovery after reversible damage to cell structures, slow removal of the antibiotic from its binding site, and the need to synthesize new enzymes before a bacterium can resume growth [17].

In this paper, we use the mathematical model of ribosome-targeting antibiotics introduced in [8] to make dynamical predictions for the response of bacterial growth rate to a time-varying antibiotic concentration. The model predicts qualitatively different dynamical responses for ribosome-targeting antibiotics that bind to ribosomes with high affinity and/or are transported into the cell irreversibly (‘high-affinity antibiotics’), as opposed to antibiotics that bind with low affinity and/or are transported reversibly (‘low-affinity antibiotics’). Our results reproduce known pharmacodynamic phenomena, such as a post-antibiotic effect for high-affinity ribosome-targeting antibiotics. Our model also predicts new phenomena, including a transient increase in growth rate upon removal of a low-affinity ribosome-targeting antibiotic. We suggest ways in which these predictions could be tested experimentally and comment on their potential clinical relevance.

## Background: mathematical model for the action of ribosome-targeting antibiotics

We first provide a brief description of the model which was introduced in [8]. The model aims to predict the growth inhibition curve: the growth rate λ of a bacterial population, as a function of the antibiotic concentration *a*_ex_ to which it is exposed. Here, the growth rate is defined by *N*(*t*) ~ exp(λ*t*), where *N*(*t*) is the number of bacteria at time *t*. The growth inhibition curve λ(*a*_ex_) is expected to be a decreasing function, which can be conveniently characterised by the concentration of antibiotic required to halve the growth rate, known as the IC_50_.

**Figure 1:**
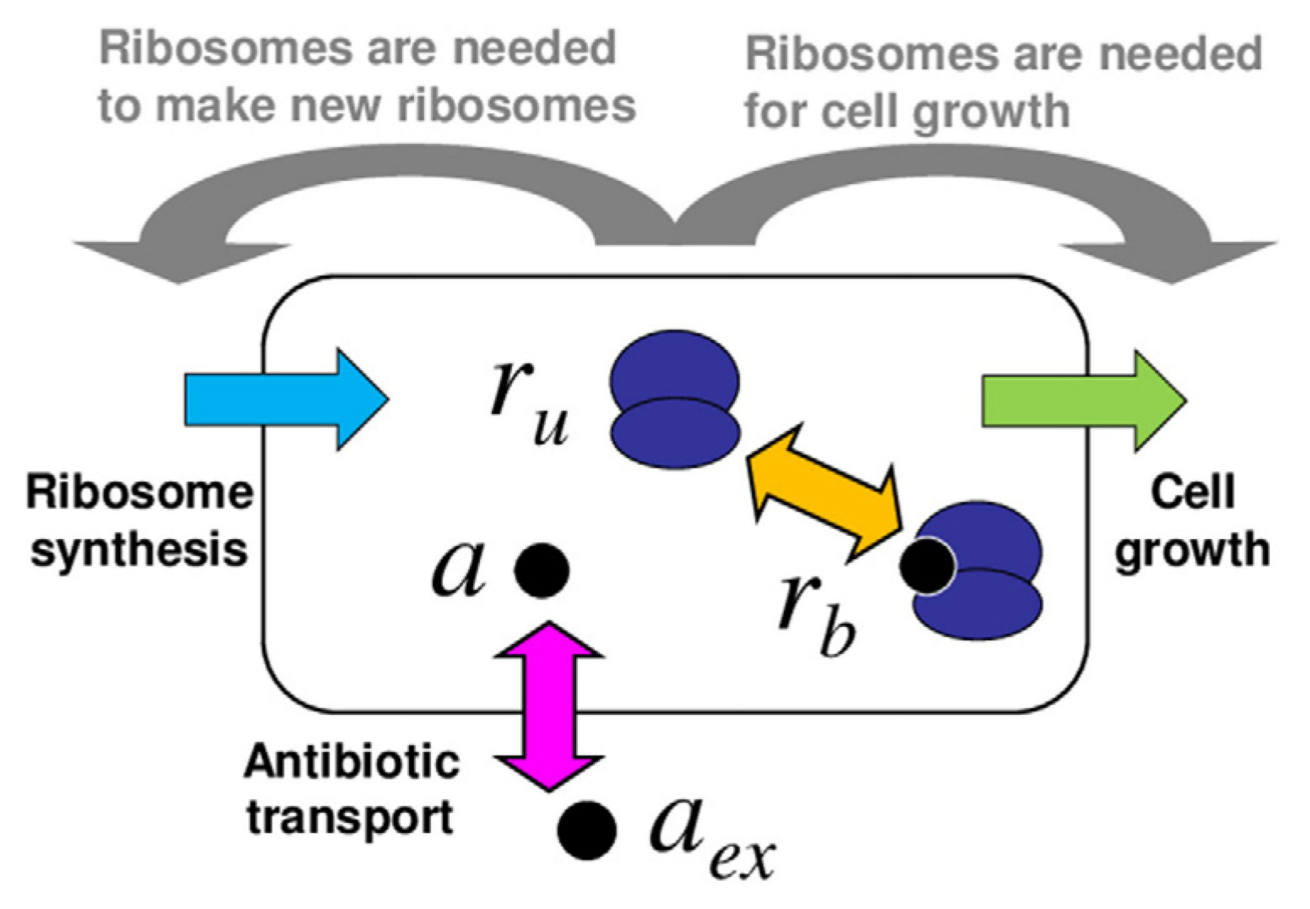
Schematic illustration of the model. The bacterial cell is modelled as a well-mixed vessel, containing ribosomes (dark blue) which may be free or bound by antibiotic. Antibiotic molecules (black circles) can be transported into or out of the cell (pink arrow) and can bind to or dissociate from ribosomes (orange arrow). The model also includes cell growth (green) and ribosome synthesis (light blue), both of which are coupled to the state of the cell (these couplings are illustrated by the grey arrows).

As illustrated in figure 1, the model describes a bacterial cell as homogeneous mixture of ribosomes and antibiotic molecules, which can bind reversibly to the ribosomes. The variables of the model are the concentrations of bound and unbound ribosomes, *r*_b_ and *r*_u_ respectively, and the concentration of intracellular antibiotic *a*. The model consists of the following set of equations for the dynamics of the concentrations:

(1) $$\dot{a}=-F\left(a,r_{\text{u}},r_{\text{b}}\right)-\lambda a+P_{\text{in}}a_{\text{ex}}-P_{\text{out}}a,$$

(2) $${\dot{r}}_{\text{u}}=-F\left(a,r_{\text{u}},r_{\text{b}}\right)-\lambda r_{\text{u}}+s,$$

(3) $${\dot{r}}_{\text{b}}=F\left(a,r_{\text{u}},r_{\text{b}}\right)-\lambda r_{\text{b}}.$$

Here, *F*(*a*, *r*_u_, *r*_b_) ≡ *k*_on_*a*(*r*_u_ − *r*_min_) − *k*_off_ *r*_b_ describes the antibiotic-ribosome binding / unbinding kinetics. *P*_in_ and *P*_out_ are rate constants (or permeabilities) for antibiotic transport into and out of the cell (both assumed to be linear processes), and *a*_ex_(*t*) is the external antibiotic concentration, which can be controlled in a lab experiment, or is set by the dosing regime in a clinical scenario. The terms −λ*a*, −λ*r*_u_ and −λ*r*_b_ describe dilution of the cell contents by growth at rate λ. Finally, *s* is the rate of synthesis of new ribosomes.

To complete the model, we need to describe how the growth rate λ and the ribosome synthesis rate *s* depend on the state of the system (grey arrows in figure 1). To do this, we use the empirical ‘growth laws’ of Scott *et al* [20, 21]. These are experimentally-established mathematical relations that describe how a bacterial cell balances the production of new ribosomes and of other proteins, depending on its growth rate. The first of these relations states that the growth rate λ is linearly related to the concentration of unbound ribosomes *r*_u_:

(4) $$r_{\text{u}}=r_{\min}+\frac{\lambda }{{\text{κ}}_{t}}.$$

Relation (4) is based on measurements in the absence of antibiotic [20, 22]; we assume here, as in [8], that it also holds in the presence of antibiotic^6^. The constant *κ_t_* = 6.1 × 10^−2^ *µ*M h^−1^ is the translation rate of the ribosomes and the constant *r*_min_ = 19.3 *µ*M is believed to arise from an inactive pool of ribosomes which may be waiting to initiate translation or stalled during translation [20, 23]. We have implicitly assumed that these inactive ribosomes do not bind antibiotic, through our definition of the binding function *F*(*a*, *r*_u_, *r*_b_)^7^.

The ribosome synthesis rate *s* can be deduced from the second ‘growth law’ of Scott *et al*, which states that the total ribosome content *r*_tot_ is linearly related to the growth rate [20]:

(5) $$r_{\text{tot}}=r_{\max}-\Delta r\lambda \left(\frac{\text{1}}{\lambda_{\text{0}}}-\frac{1}{\left({\text{κ}}_{t}\Delta r\right)}\right)$$

where *r*_max_ = 65.8 *µ*M is a universal maximal ribosome concentration [20], Δ*r* = *r*_max_ − *r*_min_ = 46.5 *µ*M is the dynamic range of the active ribosome concentration and λ_0_ is the bacterial growth rate in the absence of antibiotic. Equation (5) states that the total ribosome content increases as growth rate decreases due to ribosome inhibition: this is because of up-regulation of ribosome synthesis [24–26]. However, the slope of this increase depends on how fast the cells were growing before they were inhibited (i.e. on λ_0_). Fast-growing cells, in a rich growth medium, increase their ribosome content proportionally less than slow-growing cells, in a poor growth medium, do. Intuitively, fast-growing cells, which have a high ribosome content, already need to devote close-to-maximal protein production capacity to ribosome production so cannot increase ribosome synthesis further upon antibiotic challenge. In contrast, slow-growing cells, which have lower ribosome content, have excess protein production capacity that can be diverted to ribosome synthesis. In our model the total ribosome concentration is given by *r*_tot_ = *r*_u_ + *r*_b_. For cells growing exponentially, the contents of the cell are in a steady state, and thus the rate of ribosome synthesis must match the rate of ribosome removal by dilution: *s* = λ*r*_tot_. This leads to a quadratic expression for the synthesis rate *s* as a function of λ:

(6) $$s\left(\lambda \right)=\lambda \left[r_{\max}-\lambda \Delta r\left(\frac{\text{1}}{\lambda_{\text{0}}}-\frac{1}{\left({\text{κ}}_{t}\Delta r\right)}\right)\right].$$

Equations (1)–(3) together with (4) and (6) constitute a complete description of the model^8^.

Reference [8] focused on the stationary points of the system of equations (1)–(3), (4) and (6). Briefly, setting the time derivatives in equations (1)–(3) to zero: *ȧ* = *ṙ*_u_ = *ṙ*_b_ = 0, using equation (4) to eliminate *r*_u_ in favour of λ, then using equation (3) to eliminate *r*_b_ in equations (1) and (2) leads to two independent relations between *a* and λ, which can be solved to eliminate *a*. This leads finally to a cubic equation for the stationary points of the growth rate λ^9^:

(7) $$\begin{array}{l} 0=-{\left(\frac{\lambda }{\lambda_{0}}\right)}^{3}{\left(\frac{\lambda_{0}}{\lambda_{0}^{*}}\right)}^{2}\left[\left(1+\frac{\kappa_{t}}{k_{\text{on}}}\right)\right]\\ \;+{\left(\frac{\lambda }{\lambda_{0}}\right)}^{2}[\left(1+\frac{\kappa_{t}}{k_{\text{on}}}\right){\left(\frac{\lambda_{0}}{\lambda_{0}^{*}}\right)}^{2}\\ \;-\left(\frac{P_{\text{out}}+k_{\text{off}}}{2\sqrt{P_{\text{out}}k_{\text{off}}}}\right)\left(\sqrt{\frac{\kappa_{t}}{k_{\text{on}}}}\right)\left(\frac{\lambda_{0}}{\lambda_{0}^{*}}\right)]\\ \;+\left(\frac{\lambda }{\lambda_{0}}\right)\left[\left(\frac{P_{\text{out}}+k_{\text{off}}}{2\sqrt{P_{\text{out}}k_{\text{off}}}}\right)\left(\sqrt{\frac{\kappa_{t}}{k_{\text{on}}}}\right)\left(\frac{\lambda_{0}}{\lambda_{0}^{*}}\right)-\frac{a_{\text{ex}}}{2{\text{IC}}_{50}^{*}}\left(\frac{\lambda_{0}}{\lambda_{0}^{*}}\right)-\frac{1}{4}\right]+\frac{1}{4}, \end{array}$$

where *K*_D_ = *k*_off_ */k*_on_ and we have defined the parameter combinations $\lambda_{0}^{*}=2\sqrt{P_{\text{out}}{\text{κ}}_{t}K_{\text{D}}}$ and ${\text{IC}}_{50}^{*}=\lambda_{0}^{*}\Delta r/(2P_{\text{in}}).$

**Figure 2:**
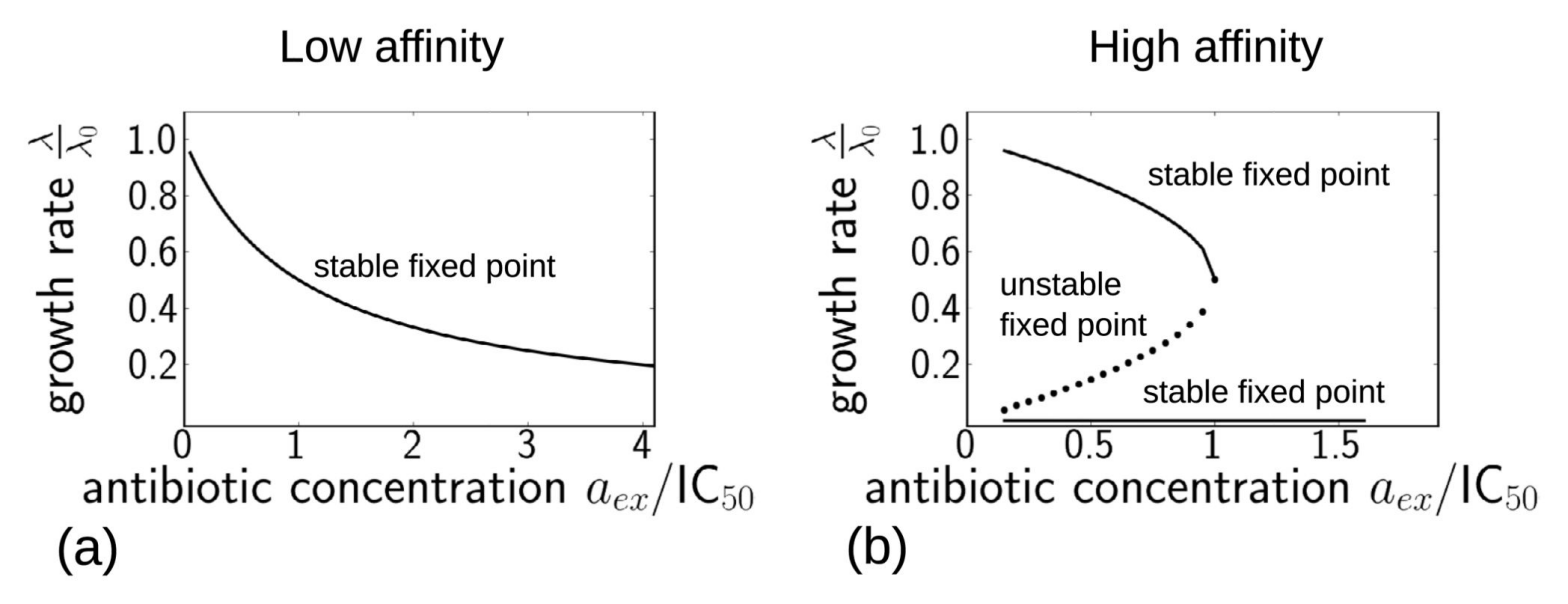
Fixed points of the model, from equation (7), plotted as a function of the external antibiotic concentration *a*_ex_, for the two parameter sets given in table 1, corresponding to antibiotics with large and small values of $\lambda_{0}^{*}$. Panel (a) shows results for a low-affinity antibiotic, with a large value of $\lambda_{0}^{*}$ (table 1). Panel (b) shows results for a high-affinity antibiotic, with a small value of $\lambda_{0}^{*}$ (table 1). The fixed points were obtained numerically in Python using sympy.solvers.solve. The external antibiotic concentration is scaled by the IC_50_ value calculated from equation (8) and given in table 1.

If the effective parameter $\lambda_{0}^{*}$ is large, corresponding to reversible antibiotic transport and/or low-affinity binding (large values of *P*_out_ and/or *K_D_* = *k*_off_ */k*_on_), then equation (7) has a single fixed point for any given value of *a*_ex_, as illustrated in figure 2(a). This corresponds to a smoothly decreasing growth inhibition curve, as observed experimentally in [8] for the antibiotics chloramphenicol and tetracycline. In contrast, if the parameter $\lambda_{0}^{*}$ is small, corresponding to irreversible transport and/or high-affinity binding (small values of *P*_out_ and/or *K_D_* = *k*_off_ */k*_on_), then the model solution has 3 fixed points for values of *a*_ex_ below a critical threshold, which is a bifurcation point (figure 2(b)). The upper and lower fixed points are stable sinks and the intermediate fixed point is an unstable saddle point of the dynamics. For example, for the parameter set of figure 2(b), for *a*_ex_ = 0.9 ×IC_50_ (below the bifurcation point), the real parts of the eigenvalues of the Jacobean matrix at the three fixed points are (−1.1 × 10^4^, −0.66, −0.90), (−5.7 × 10^3^, 0.89, −0.34), (−1.0 × 10^6^, −1.0 × 10^−3^, −2.7 × 10^−5^). Thus, the first and third fixed points are stable (their eigenvalues have all negative real parts) while the second fixed point is unstable (it has an eigenvalue with a postive real part). For *a*_ex_ = 1.1 × IC_50_ (above the bifurcation point), the single fixed point has eigenvalues with all negative real parts (−1.3 × 10^6^, −1.0 × 10^−2^, −2.2 × 10^−5^) - i.e. it is stable. The bifurcation points of the model are discussed in more detail in appendix A, where it is also argued that the value $a_{\text{ex}}^{*}=\Delta r\lambda_{0}/(4P_{\text{in}})$ is a good approximation for the (upper) bifurcation point. From a practical point of view, if $\lambda_{0}^{*}$ is small, then for small values of the external antibiotic concentration, we expect to observe little inhibition of bacterial growth, corresponding to the upper fixed point of the dynamics. However, for antibiotic concentrations above the bifurcation point, we expect to see drastic growth inhibition, corresponding to the single (lower) fixed point. This implies a steep, threshold-like growth inhibition curve, as observed experimentally in [8] for the aminoglycosides streptomycin and kanamycin^10^.

Equation (7) can also be used to derive a simple expression for the dependence of the IC_50_ on λ_0_ [8]:

(8) $$\begin{array}{l} \frac{{\text{IC}}_{50}}{{\text{IC}}_{50}^{*}}=\frac{1}{2}[\left(1+\frac{\kappa_{t}}{k_{\text{on}}}\right)\left(\frac{\lambda_{0}}{\lambda_{0}^{*}}\right)+\left(\frac{\lambda_{0}^{*}}{\lambda_{0}}\right)\\ \;+\frac{\left(P_{\text{out}}+k_{\text{off}}\right)}{\sqrt{P_{\text{out}}k_{\text{off}}}}\sqrt{\frac{\kappa_{t}}{k_{\text{on}}}}]. \end{array}$$

Equation (8) predicts that antibiotic efficacy will increase with nutrient richness (IC_50_ decreases with λ_0_) when $\lambda_{0}^{*}$ is large, but that efficacy will decrease with nutrient richness (IC_50_ increases with λ_0_) when $\lambda_{0}^{*}$ is small. These predictions, which are in agreement with experimental data, were discussed in detail in [8].

## Results: model predictions for dynamical response to antibiotic

In a clinical context, antibiotic concentrations vary in time. In this paper, we explore the predictions of the model defined by equations (1)–(3), (4) and (6), for the response of bacterial growth rate to a time-dependent exposure to antibiotic—i.e. we explore the dynamics *a*(*t*), *r*_u_(*t*) and *r*_b_(*t*) for a time-varying external antibiotic concentration *a*_ex_(*t*). In most cases (with some exceptions that we discuss below), these equations are not amenable to an analytical solution in the time-varying case.We therefore integrate the model equations numerically, starting from the steady-state solution in the absence of antibiotic^11^. We compare results for two sets of parameters, representing antibiotics which are bound and transported with ‘low affinity’ (high values of *P*_out_/*P*_in_ and *k*_off_ */k*_on_) and with ‘high affinity’ (low values of *P*_out_*/P*_in_ and *k*_off_ */k*_on_). These parameters, which are chosen to be within the range of literature values for tetracycline and streptomycin respectively^12^, are listed in table 1.

It is important to note that the growth laws that we use in our model, equations (4) and (6), are derived from experimental measurements on exponentially growing bacteria, for which all intracellular concentrations are in steady state. In using these constraints to make predictions for dynamical trajectories we assume that the cell adjusts its rates of growth and ribosome synthesis rapidly in response to changing external conditions, in comparison to the rate at which the external conditions vary. It is known that the ribosome synthesis rate can adjust within minutes to changes in nutrient conditions [27]. A typical timescale for synthesis of a protein molecule is ~1 minute (~1000 amino acids polymerised at a translation rate of ~20 amino acids per second [22]), while a conservative estimate for the timescale for synthesis of a ribosome is ~6 min (~7500 amino acids in the entire ribosomal complex, produced at ~20 amino acids per second [22]). The timescale over which antibiotic concentration builds up in the body after an oral dose is ~30 minutes, with a slower decay time due to excretion [15]. The use of the steady-state constraints (4) and (6) therefore seems reasonable.

### Response to a step increase in antibiotic

To analyse the dynamical behaviour of the model, we first consider the response to a sudden, step-like increase in antibiotic concentration, from zero to a fixed value: *a*_ex_(*t*) = 0 for *t* < *t*_0_ and $a_{\text{ex}}\left(t\right)=a_{\text{ex}}^{\text{final}}$ for *t* > *t*_0_. In the clinical context, this would correspond to an intravenous infusion of antibiotic; in the laboratory it could be achieved using a continuous culture device [28, 29] or microfluidic flow device [30]).

#### Low-affinity antibiotic

**Figure 3:**
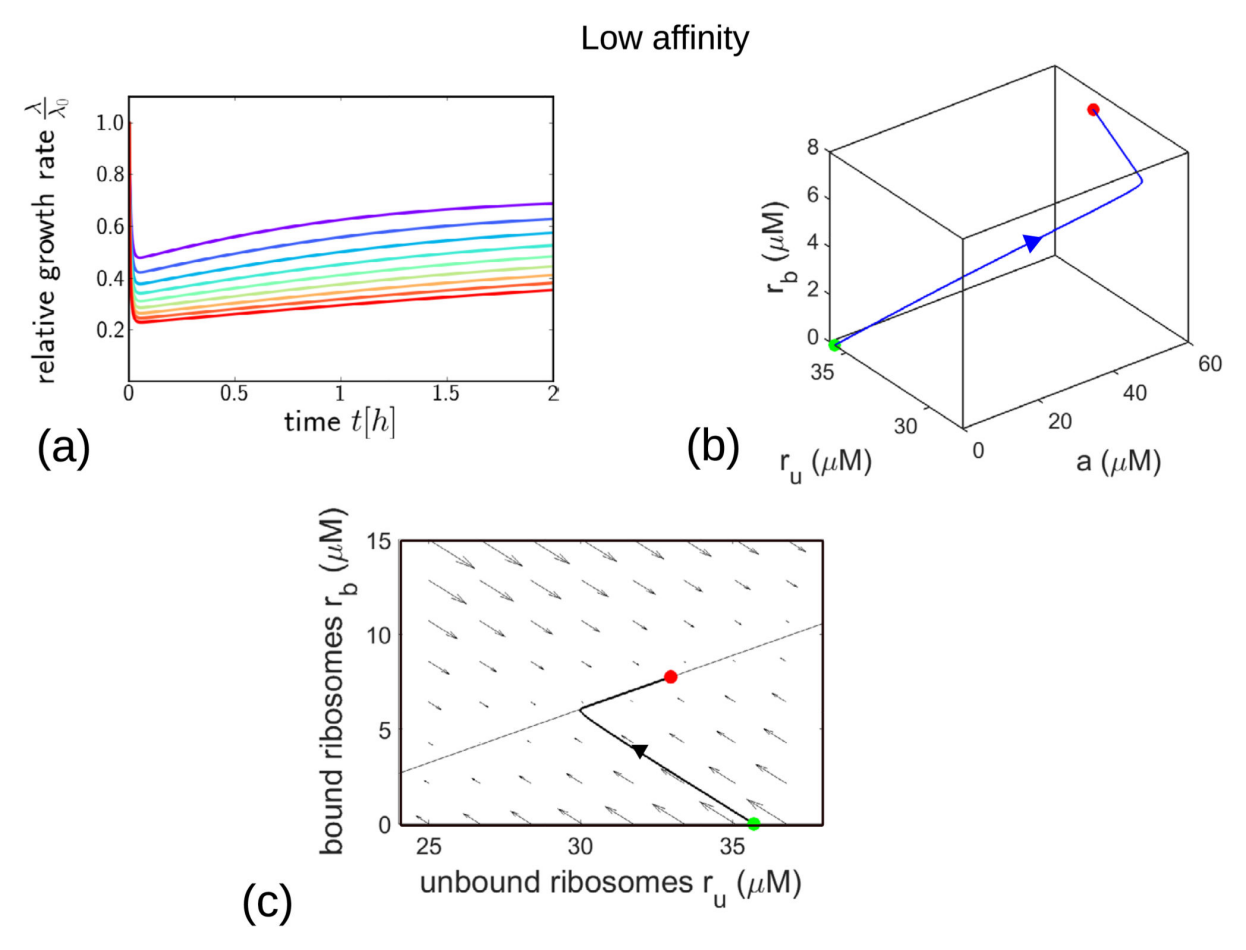
Dynamical trajectories showing growth inhibition after a step increase in antibiotic concentration, for the low-affinity parameter set (with parameter values as in table 1). (a) Relative growth rate λ(*t*)/λ_0_ as a function of time after the step increase in antibiotic concentration. The final antibiotic concentration $a_{\text{ex}}^{\text{final}}$ is indicated by the line colour, ranging from 0.4 ×IC_50_ (purple) to1.3 ×IC_50_ (red), in steps of 0.1 ×IC_50_. (b) Trajectory in the 3-dimensional space of variables *a*, *r*_u_ and *r*_b_, for the case of final a step increase to $a_{\text{ex}}^{\text{final}}=2\times {\text{IC}}_{\text{50}}$. The initial and final system states are shown by the green and red points respectively. (c) Flow diagram showing the direction and magnitude of the flow field *ṙ*_u_, *ṙ*_b_ for a fixed value of *a* corresponding to the final point of the trajectory in panel (b), and for *a_ex_* = *2* ×IC_50_. The thin solid line shows the nullcline corresponding to *ṙ*_u_ = 0; this is given by $k_{\text{off}}r_{b}=\left(r_{\text{u}}-r_{\min}\right)\left(k_{\text{on}}a-{\text{κ}}_{t}\Delta r\right)+{\left(r_{\text{u}}-r_{\min}\right)}^{2}{\text{κ}}_{t}^{\text{2}}\Delta r/\lambda_{\text{0}}.$ The other nullcline, defined by *ṙ*_b_ = 0, is not shown here. The trajectory from (b), projected onto the *r*_u_, *r*_b_ plane, is shown as the thicker solid line.

Figure 3 explores the dynamical response of the model to a step increase in concentration of a low-affinity antibiotic. The model predicts a strikingly non-monotonic response of the bacterial growth rate λ(*t*)/λ_0_, as shown in figure 3(a): we observe an initial rapid decrease in growth rate, followed by a slower recovery to a steady-state value that depends on the antibiotic concentration $a_{\text{ex}}^{\text{final}}$. This steady-state value corresponds to the fixed point of the model dynamics (figure 2(a)). The origin of this non-monotonic response can be understood by plotting the trajectory of the model in the 3d space of its variables *a*, *r*_u_ and *r*_b_, as in figure 3(b). Following the increase in *a*_ex_, the intracellular antibiotic concentration *a* rapidly increases, accompanied by a decrease in the concentration of unbound ribosomes and an increase in the concentration of bound ribosomes *r*_b_. These changes are driven by the rapid dynamics of antibiotic transport and binding/unbinding. The later, much slower, recovery of the growth rate observed in figure 3(a) corresponds to an increase in both *r*_u_ and *r*_b_ in the trajectory of figure 3(b) and is associated with the slower dynamics of ribosome synthesis in response to the antibiotic challenge. Thus, the non-monotonic response of the growth rate predicted by the model is due to the initial, rapid processes of transport and binding, followed by a slower partial recovery due to increased ribosome synthesis.

The dynamics of the model can also be illustrated in the form of a flow diagram, as in figure 3(c). Here, the arrows show the direction of the flow field *ṙ*_u_, *ṙ*_b_ for a fixed value of *a*, while the solid line shows the nullcline *ṙ*_u_ = 0. The trajectory of figure 3(b) is shown projected onto this plane. This diagram illustrates clearly the separation of timescales between transport and binding, which produces a strong flow field towards the centre of the diagram, and ribosome synthesis, which is responsible for the slower dynamics along the nullcline as the system approaches the stable fixed point.

#### High-affinity antibiotic

**Figure 4:**
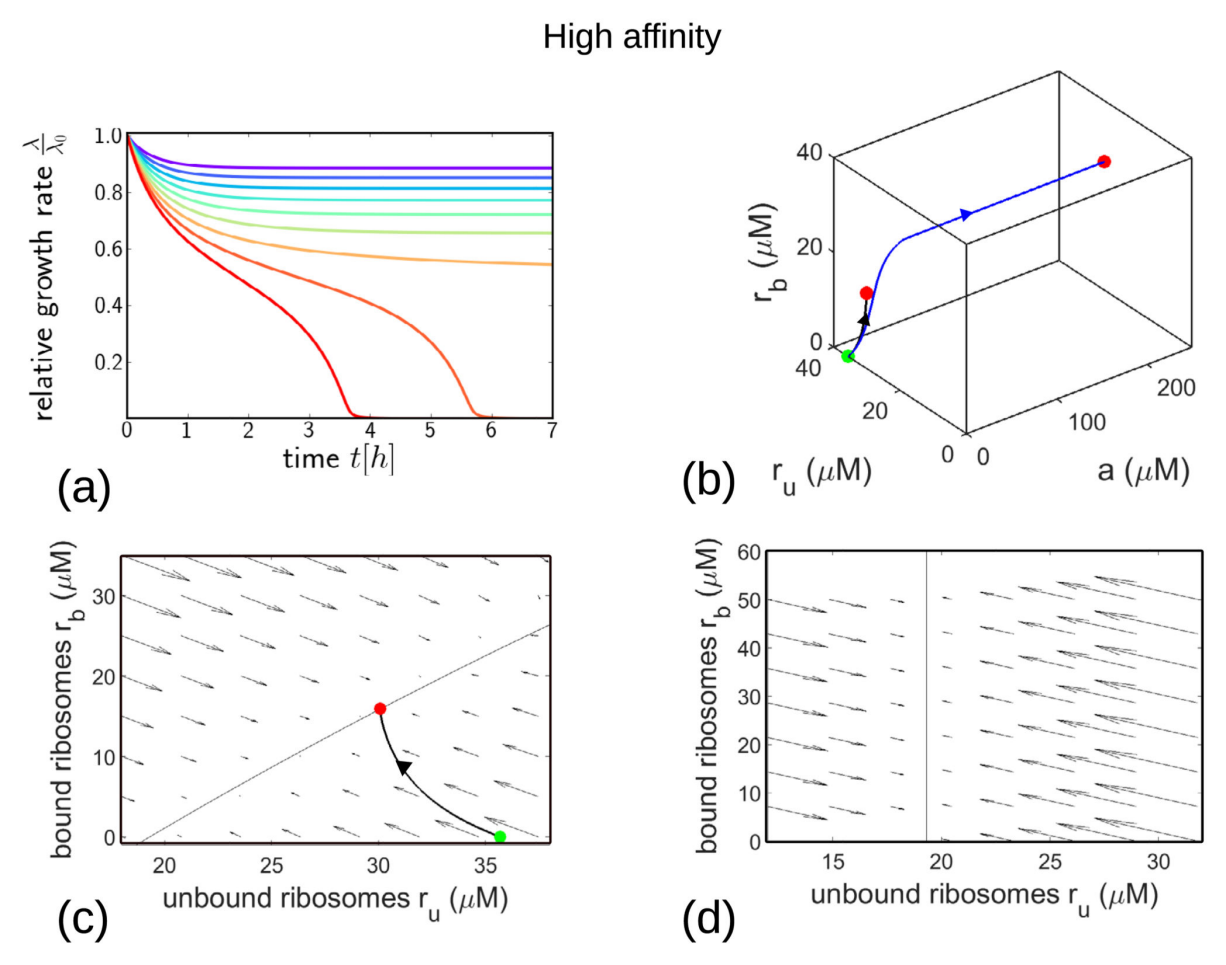
Dynamical trajectories showing growth inhibition after a step increase in antibiotic concentration, for the high-affinity parameter set (with parameter values as in table 1). (a): Relative growth rate λ(*t*)/λ_0_ as a function of time after the step increase in antibiotic concentration. The final antibiotic concentration $a_{\text{ex}}^{\text{final}}$ is indicated by the line colour, ranging from 0.4 × IC_50_ (purple) to 1.3 × IC_50_ (red), in steps of 0.1 × IC_50_. (b): Trajectory in the 3-dimensional space of variables *a*, *r*_u_ and *r*_b_, for the case of a step increase to $a_{\text{ex}}^{\text{final}}=0.9\times {\text{IC}}_{\text{50}}$ (black line) and $a_{\text{ex}}^{\text{final}}=1.1\times {\text{IC}}_{\text{50}}$ (blue line). The initial and final system states are shown by the green and red points respectively. (c): Flow diagram showing the direction and magnitude of the flow field *ṙ*_u_, *ṙ*_b_ for a fixed value of *a* corresponding to the final point of the black trajectory in panel (b), and for *a*_ex_ = 0.9 ×IC_50_. The thin solid line shows the nullcline *ṙ*_u_ = 0, given by $k_{\text{off}}r_{b}=\left(r_{\text{u}}-r_{\min}\right)\left(k_{\text{on}}a-{\text{κ}}_{t}\Delta r\right)+{\left(r_{\text{u}}-r_{\min}\right)}^{2}{\text{κ}}_{t}^{\text{2}}\Delta r/\lambda_{\text{0}}.$ The trajectory from (b), projected onto the *r*_u_, *r*_b_ plane, is shown as the thick solid line. (d): Flow diagram showing the flow field *ṙ*_u_, *ṙ*_b_ for a fixed value of *a* = 200 *µ*M and for *a*_ex_ = 0.9 ×IC_50_. The nullcline *ṙ*_u_ = 0 is shown by the thin solid line.

The model predictions are strikingly different for the high-affinity antibiotic (figure 4). The bacterial growth rate (figure 4 (a)) is predicted to decrease smoothly and monotonically for low antibiotic concentrations $a_{\text{ex}}^{\text{final}}$ as it approaches the upper fixed point in figure 2(b). However, for antibiotic concentrations $a_{\text{ex}}^{\text{final}}$ final that are above the bifurcation point in figure 2(b) the model predicts instead a decline in growth rate to a state in which there is essentially no growth. The timescale of this approach to the non-growing steady state can be very long (of the order of days) for antibiotic concentrations close to the bifurcation point.

Plotting the dynamics of the model in the 3d space of its variables (figure 4(b)) illustrates the very different nature of its trajectories for values of $a_{\text{ex}}^{\text{final}}$ below and above the bifurcation point. If $a_{\text{ex}}^{\text{final}}$ is below the bifurcation point, as for the black trajectory in figure 4(b) $\left(\text{for}\;a_{\text{ex}}^{\text{final}}=0.9\times {\text{IC}}_{\text{50}}\right),$ the dynamics approaches a fixed point which is close to the initial state, and in which the intracellular antibiotic concentration is small. This corresponds to the upper stable fixed point in figure 2(b). However, if $a_{\text{ex}}^{\text{final}}$ is above the bifurcation point, as for the blue trajectory $\left(\text{for}\;a_{\text{ex}}^{\text{final}}=1.1\times {\text{IC}}_{\text{50}}\right),$ the dynamics instead approaches a very different state, with a far higher intracellular antibiotic concentration and with *r*_u_ close to *r*_min_ = 19.3 *µ*M: this corresponds to the lower fixed point in figure 2(b), with essentially no growth.

The flow diagrams of figures 4(c) and (d) illustrate the two stable fixed points of the model dynamics for values of *a*_ex_ below the bifurcation point. These diagrams show the flow field *ṙ*_u_, *ṙ*_b_ for *a*_ex_ = 0.9 × IC_50_, for two different values of the intracellular antibiotic concentration *a*. In figure 4(c), the flow field is shown for *a* = 0.016 *µ*M, which corresponds to the final point of the black trajectory in figure 4(b) (this trajectory is also shown, projected onto the *a* = 0.016 *µ*M plane). The model has a stable fixed point for values of *r*_u_ and *r*_b_ which are close to the starting point of the trajectory (i.e. the system state in the absence of antibiotic). The second stable fixed point is evident in figure 4(d), which shows the flow field for *a* = 200 *µ*M. This fixed point occurs at a much smaller value of *r*_u_ (*r*_u_ ≈ *r*_min_) and a higher value of *r*_b_. Interestingly, as for the low-affinity case (figure 3(c)), the flow diagrams of figure 4 show a separation of timescales between the rapid dynamics of transport and binding and the slower dynamics of ribosome synthesis. However the separation is less extreme than for the low-affinity case (since *P*_in_, *P*_out_ and *k*_off_ are all smaller)—this may explain why the approach to the stable state is monotonic rather than non-monotonic for our high-affinity parameter set.

**Figure 5:**
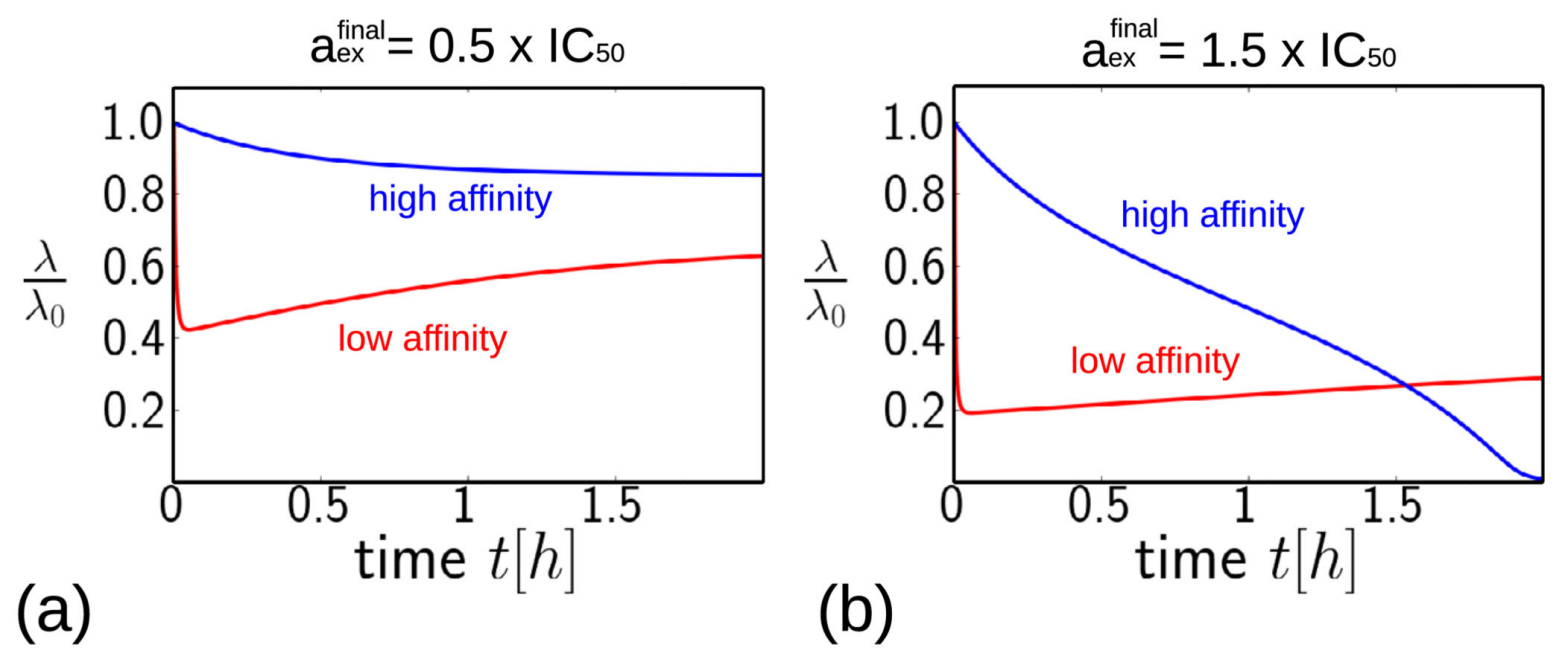
Dynamical trajectories showing growth inhibition after a step increase in antibiotic concentration, for the high-affinity and low-affinity parameter sets (blue and red lines, respectively, with parameter values as in table 1). (a) Predictions for a step increase to a final antibiotic concentration equal to half the IC_50_. (b) Equivalent predictions for a final antibiotic concentration equal to1.5 ×IC_50_. Note that the IC_50_ values are calculated as in table 1, and are different for the low and high-affinity antibiotics.

Figure 5 compares directly the predictions of the model for a step increase in antibiotic concentration, for the low-affinity antibiotic (shown in red) and the high-affinity antibiotic (shown in blue), for antibiotic concentrations $a_{\text{ex}}^{\text{final}}=0.5\times {\text{IC}}_{\text{50}}$ and $a_{\text{ex}}^{\text{final}}=1.5\times {\text{IC}}_{\text{50}}$. The low-affinity antibiotic produces faster growth inhibition than the high-affinity antibiotic. For concentrations of antibiotic below the IC_50_ (figure 5(a)), the low-affinity antibiotic also achieves stronger inhibition at long times, for the ‘equivalent’ concentration. However, for antibiotic concentrations above the IC_50_ (figure 5(b)), the final degree of inhibition is greater for the high-affinity antibiotic. This difference in final inhibition level arises from the behaviour of the fixed points of the model as a function of antibiotic concentration (figure 2). For concentrations below the IC_50_, the stable fixed point for the low-affinity parameter set has a lower growth rate than that for the high-affinity parameter set, but for concentrations above the IC_50_, the situation is reversed (figure 2, compare (a) and (b)).

### Time to full inhibition

From a practical point of view, it is important to know the time required to achieve maximal growth inhibition following an antibiotic dose. Because the variables in our model are continuous, the growth rate never completely reaches zero, but as a proxy for full inhibition we measure the time taken to achieve 99% inhibition, i.e. to reduce the growth rate to 1% of its antibiotic-free value: λ/λ_0_ = 0.01. Although this is an arbitrary threshold, in a clinical situation a drastic reduction in bacterial population density is expected to lead to elimination of an infection, due to the action of the immune system [31]. In our model, 99% inhibition only occurs for higher concentrations of antibiotic; for lower concentrations the system instead reaches a steady state with a growth rate greater than 0.01 × λ_0_ (as shown in figures 3(a) and 4(a)).

**Figure 6:**
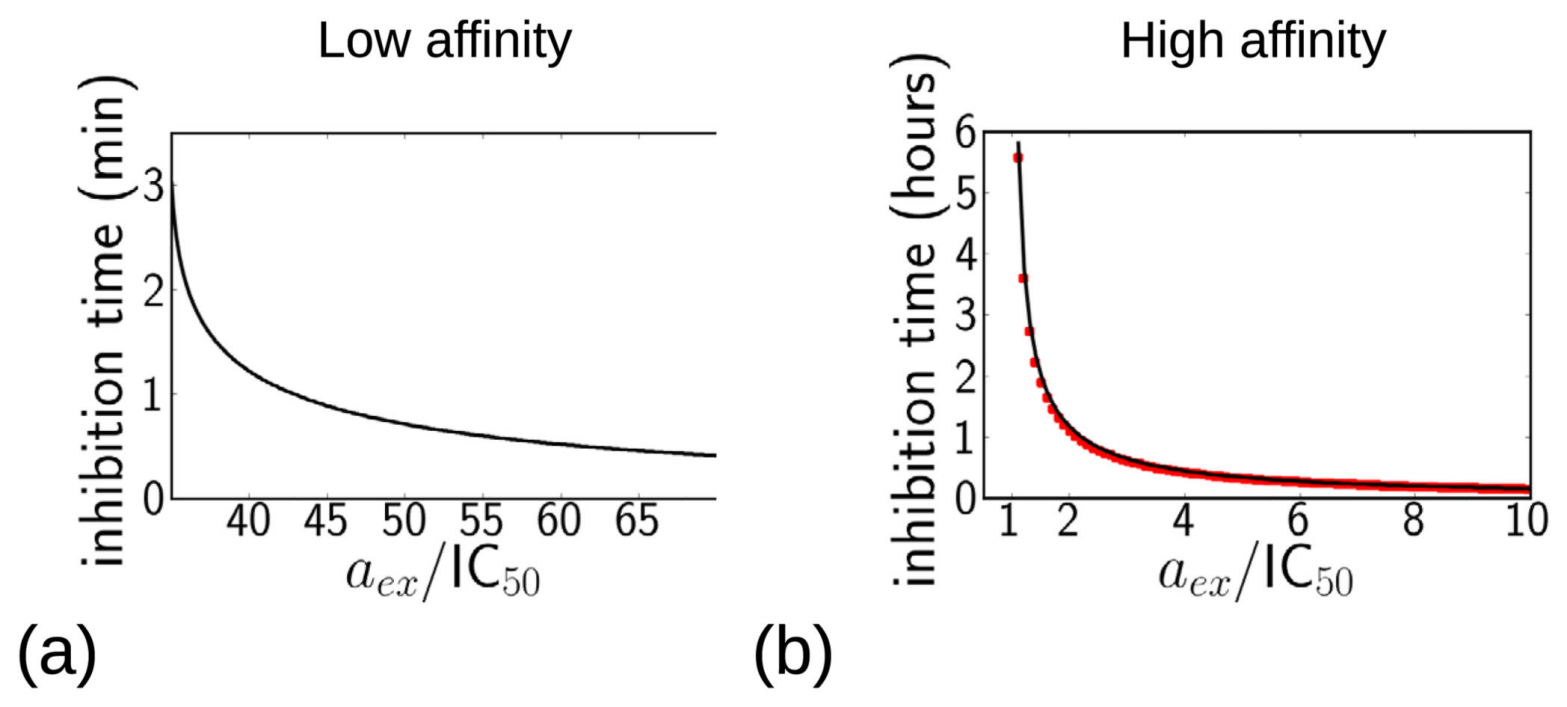
Model predictions for the time required to reach 99% growth inhibition λ/λ_0_ = 0.01, following a step increase in antibiotic concentration. The inhibition time is plotted as a function of the external antibiotic concentration (note that the time is measured in minutes in (a) but in hours in (b)). Panel (a) shows results for the low-affinity antibiotic; panel (b) shows results for the high-affinity antibiotic (using parameters as in table 1); here the numerical solution of the model is shown by the solid line while the red symbols show the analytical prediction based on the adiabatic approximation, described in appendix B. For antibiotic concentrations lower than those plotted here, the dynamical trajectory of λ/λ_0_ always stays above 0.01.

Figure 6 shows the time to reach λ = 0.01 × λ_0_, as a function of the antibiotic concentration, for the low-affinity and high-affinity antibiotics. As expected, higher antibiotic concentrations lead to more rapid growth inhibition. For the low-affinity antibiotic (figure 6(a)), a high concentration is needed to achieve 99% inhibition (~35 ×IC_50_), but for these concentrations, 99% inhibition is achieved very rapidly, on a timescale of minutes, and the inhibition time decreases smoothly as the antibiotic concentration increases. This is consistent with the inhibition trajectories shown in figure 3(a), which show a rapid initial inhibition of growth. For the high-affinity antibiotic (figure 6(b)), 99% inhibition is achieved for much lower concentrations of antibiotic, just above the IC_50_, but the timescale for inhibition is longer, of the order of hours for concentrations close to the IC_50_. This is consistent with the inhibition trajectories of figure 4(a), which show very long timescales for inhibition for antibiotic concentrations close to the bifurcation point of the model dynamics. As we discuss in appendix B (and illustrate in figure B1), this very slow inhibition occurs because of a ‘bottleneck’ effect, in which dynamical trajectories slow down as they pass close to the location where the two fixed points have merged.

For the high-affinity antibiotic, it is possible to obtain an analytical prediction for the time to achieve 99% inhibition, by making an adiabatic approximation for the dynamics of the intracellular antibiotic concentration *a*(*t*). This calculation is presented in detail in appendix B; briefly, we assume that the dynamics of *a*(*t*) are fast compared to those of the other variables, and set *ȧ* = 0 in equations (1)–(3). This reduces the model to a set of dynamical equations for *r*_u_ and *r*_b_, and setting *k*_off_ = 0 (for an irreversible antibiotic) decouples these equations, allowing one to solve for *r*_u_, and hence for the growth rate λ(*t*) via the constraint (4). Figure 6(b) shows that the resulting analytical prediction for the inhibition time (red symbols) is in good agreement with the numerical results (black solid line).

### Response to a step pulse of antibiotic

**Figure 7:**
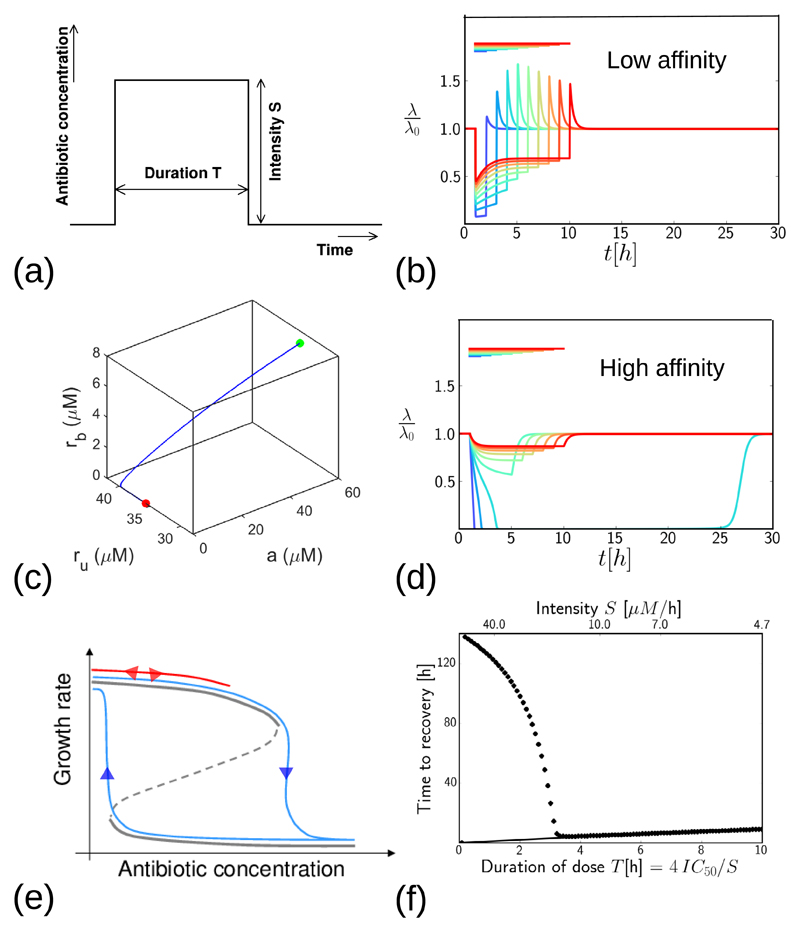
Growth inhibition in response to a transient step-like dose of antibiotic. (a) Illustration of the dosing protocol: antibiotic concentration is switched suddenly to a value *S* at the start of the dose, and is switched back to zero after a time *T*. The total dose *S* × *T* is fixed at 4 × IC_50_. (b) Growth-rate trajectories for the low-affinity antibiotic (with parameters as in table 1). The coloured lines represent doses of different duration and intensity (keeping the total dose fixed at 4 × IC_50_). The colour bars show the duration of the dose. The green and red dots correspond to the start and end points of the trajectory in panel (c). (c): Trajectory in the 3-dimensional space of variables *a*, *r*_u_ and *r*_b_, after removal of the low-affinity antibiotic, for *S* = 2 × IC_50_. The green point corresponds to the time immediately before the antibiotic is removed and the red point corresponds to a much later time (*t* → ∞) - as shown in panel (b). (d) Growth-rate trajectories as in panel (b), but for the high-affinity antibiotic (with parameters as in table 1). (e) Schematic illustration of hysteresis in the model for the high-affinity antibiotic. The response to a low-intensity pulse of antibiotic is shown by the red line: upon addition of antibiotic the system tracks the upper stable fixed point and reverses its trajectory when the antibiotic is removed. The response to a high-intensity pulse is shown by the blue lines: upon addition of antibiotic the system transitions to the lower stable fixed point, and it tracks the lower fixed point when antibiotic is removed. (f) Time taken to recover from a step dose of antibiotic, as a function of the duration of the dose. The solid line shows results for the low-affinity antibiotic, the symbols show results for the high-affinity antibiotic. Here, ‘recovery’ is defined to mean that the growth rate λ returns to a value 0.9 × λ_0_, having previously fallen below this threshold. The recovery time is defined as the total time during which the growth rate is suppressed below 0.9 × λ_0_.

In a clinical situation, antibiotic treatment has a finite duration. The antibiotic concentration at the infection site increases after a dose is given and later decreases due to removal of the antibiotic from the body (the pharmocokinetic curve [15]). To mimic this, we investigate the response of the model to pulses of antibiotic of finite duration. We obtain predictions for the dynamics of the bacterial growth rate during and after the dose, for low- and high-affinity antibiotics, and for different dose durations and intensities. For simplicity, we first consider a step pulse of antibiotic of intensity *S* that is maintained for a fixed time *T*, as illustrated in figure 7(a); later we also consider a more clinically realistic scenario where the antibiotic is removed more gradually. We compare results for a fixed total antibiotic dose (duration × intensity)—i.e. we compare the effect of a short, high-intensity dose with that of a long, low-intensity dose. Specifically, we fix *S* × *T* = 4 × IC_50_. Although this choice is arbitrary, we find qualitatively similar results for other values of the total dose.

#### Low-affinity antibiotic: growth-rate overshoot following antibiotic removal

Figure 7(b) shows model predictions for the bacterial growth rate, during and after a step-like pulse of a low-affinity antibiotic. The colours indicate doses of varying duration (as shown by the bars). During the dose, bacterial growth is suppressed, to a degree that depends on the intensity of the dose (the short, high intensity dose shown by the blue line causes a greater degree of growth inhibition than the long, low intensity dose shown by the red line). Interestingly, the model also predicts a ‘growth rate overshoot’ phenomenon: a peak in λ(*t*) after the antibiotic dose ends, implying a transient increase in growth rate *above* the antibiotic-free steady-state value λ_0_. The overshoot occurs because, in our model, ribosome synthesis is upregulated during exposure to the antibiotic (*s* is larger, according to equation (6)), such that the total ribosome concentration becomes higher than it would be in the absence of antibiotic. Once the external antibiotic is removed, intracellular antibiotic dissociates rapidly from bound ribosomes, since *k*_off_ ≫ λ_0_, so that the free ribosome pool becomes transiently larger than it would have been in the absence of antibiotic. In our model, this produces a transient increase in growth rate. This is illustrated in figure 7(c), which shows a trajectory in the 3d space {*a*, *r*_u_, *r*_b_} after removal of the antibiotic, for a pulse with intensity *S* = 2 ×IC _50_. The transient increase in unbound ribosome *r*_u_ (and hence in growth rate) is coupled to loss of intracellular antibiotic *a* and bound ribosomes *r*_b_. The later decrease in *r*_u_ back to the drug-free steady state value (red dot in figure 7(c)) happens along the *r*_u_ axis, once *a* and *r*_b_ have both reached zero.

The magnitude of the transient growth-rate increase shown in 7(b) is greatest at intermediate antibiotic dose duration; this is because for very short antibiotic pulses, the bacterium does not have time to increase its ribosome pool significantly before the pulse ends, while for very long, low intensity pulses the antibiotic concentration is not high enough to produce a significant upregulation of ribosome concentration. Consistent with this explanation, when we repeat our simulations keeping the dose intensity fixed (i.e. increasing total dose as the duration increases), we find that the maximal overshoot occurs for the longest dose duration (data not shown).

Upregulation of ribosome synthesis upon exposure to antibiotic is a growth medium-dependent phenomenon: for bacteria growing in a poor medium (with a small drug-free growth rate λ_0_), the relative increase of the ribosome synthesis rate is larger than for bacteria growing on rich medium (with a large λ_0_) [20]. This is captured by the λ_0_-dependence of the synthesis rate *s* in our model (equation (6)). We therefore expect that the magnitude of the growth-rate overshoot predicted by the model will be medium-dependent, with a larger overshoot for bacteria growing on poor medium, which upregulate ribosome synthesis more strongly and therefore have a greater excess of ribosomes after the pulse. Indeed, upon repeating our calculations for a range of values of λ_0_, we observe a strong λ_0_-dependence of the magnitude of the overshoot. For example, for a dose of duration *σ* = 7 h, the growth rate at the peak of the overshoot is predicted to be λ/λ_0_ = 2.3, 1.7, 1.3, for drug-free growth rates of λ_0_ = 0.5, 1.0, 1.5 h^−1^ respectively.

#### High-affinity antibiotic: post-antibiotic growth suppression and hysteresis

Figure 7(d) shows equivalent predictions for the growth-rate response to a step pulse of high-affinity antibiotic. Here we observe a different phenomenon: the qualitative nature of the response is intensity-dependent. For long-duration, low-intensity doses the growth rate is suppressed during the dose but recovers quickly when the antibiotic is removed (red-green curves in figure 7(d)). However, for shorter, high intensity doses, the model shows a significant post-antibiotic effect: the growth rate decreases almost to zero during the dose and does not recover until many hours after the dose has ended (blue curves in figure 7(d)). This phenomenon arises from hysteresis in the model. When antibiotic is added, the fixed points of the model move along the *a*_ex_ axis in figure 2(b). As illustrated in figure 7(e), for a low-intensity antibiotic dose, the system tracks the upper stable fixed point and reverses its trajectory when the antibiotic is removed (red line in figure 7(e)). This corresponds to the red-green trajectories in figure 7(d). However, for a high-intensity antibiotic dose, the system is pushed past the bifurcation point in figure 2(b), forcing it to transition to the lower stable fixed point in which the growth rate is close to zero. When the antibiotic is removed, the system moves back along the lower line of fixed points, before eventually transitioning back to the upper fixed point (blue lines in figure 7(e)). The timescale over which this eventual recovery happens is controlled by the antibiotic-ribosome dissociation rate constant *k*_off_, which is small for the high-affinity antibiotic. Although we always see eventual recovery of the bacterial growth rate in our simulations, in a clinical setting we expect that other factors, such as immune response, would lead to elimination of the infection [31].

#### Optimal dosing strategy differs for low and high-affinity antibiotics

In a clinical setting, antibiotic dosing protocols target different features of the pharmacokinetic curve: some are designed to maximise the peak antibiotic concentration, while others aim to maximise the time the concentration is above a threshold, or the area of the curve above the threshold [15, 32]. Although our simulated step-like dosing protocol (figure 7(a)) is simplistic, we do see clear differences in optimal dosing strategy for low-affinity and high-affinity antibiotics. These differences are illustrated in figure 7(f), where we plot the time required for the bacterial growth rate to recover from a step-like antibiotic dose, as a function of the duration of the dose (and hence its inverse intensity, as shown on the upper horizontal axis). Here, we define time to recovery as the time taken for the growth rate λ to recover to 90% of its antibiotic-free steady state value λ_0_, having previously fallen to below this value. For the low-affinity antibiotic (figure 7(f), solid line), the recovery time is proportional to dose duration: this is consistent with the growth inhibition trajectory (figure 7(b)), in which growth is suppressed during the dose and recovers rapidly afterwards. Therefore, for ribosome-targeting antibiotics which bind with low affinity and/or are transported reversibly, the model suggests that an optimal protocol would maximise the time over which the dose is maintained above a threshold. This is consistent with the fact that tetracycline antibiotics, which fall into the low affinity class in [8], are categorized in the clinical pharmacodynamic literature as time-dependent, i.e. the duration of the dosage controls efficacy of treatment [33]. In contrast, for the high-affinity antibiotic (figure 7(f), symbols), the model predicts that the recovery time increases dramatically, to many times longer than the dose, when the dose intensity exceeds a well-defined threshold (i.e. for shorter dose durations in our simulations). This is also consistent with the growth inhibition trajectories of 7(d). Thus our model suggests that for ribosome-targeting antibiotics which bind with high affinity and/or are transported irreversibly, it may be more important to maximise the peak concentration of the pharmacokinetic curve than the duration of the dose. This prediction is consistent with the fact that aminoglycoside antibiotics, which fall into the high-affinity class in [8], are categorized clinically as concentration-dependent, i.e. the peak concentration controls the treatment efficacy [33]. Our predictions are also consistent with the fact that aminoglycosides can show significant post-antibiotic effects [17–19, 32, 34].

### Response to a more realistic pulse of antibiotic

**Figure 8:**
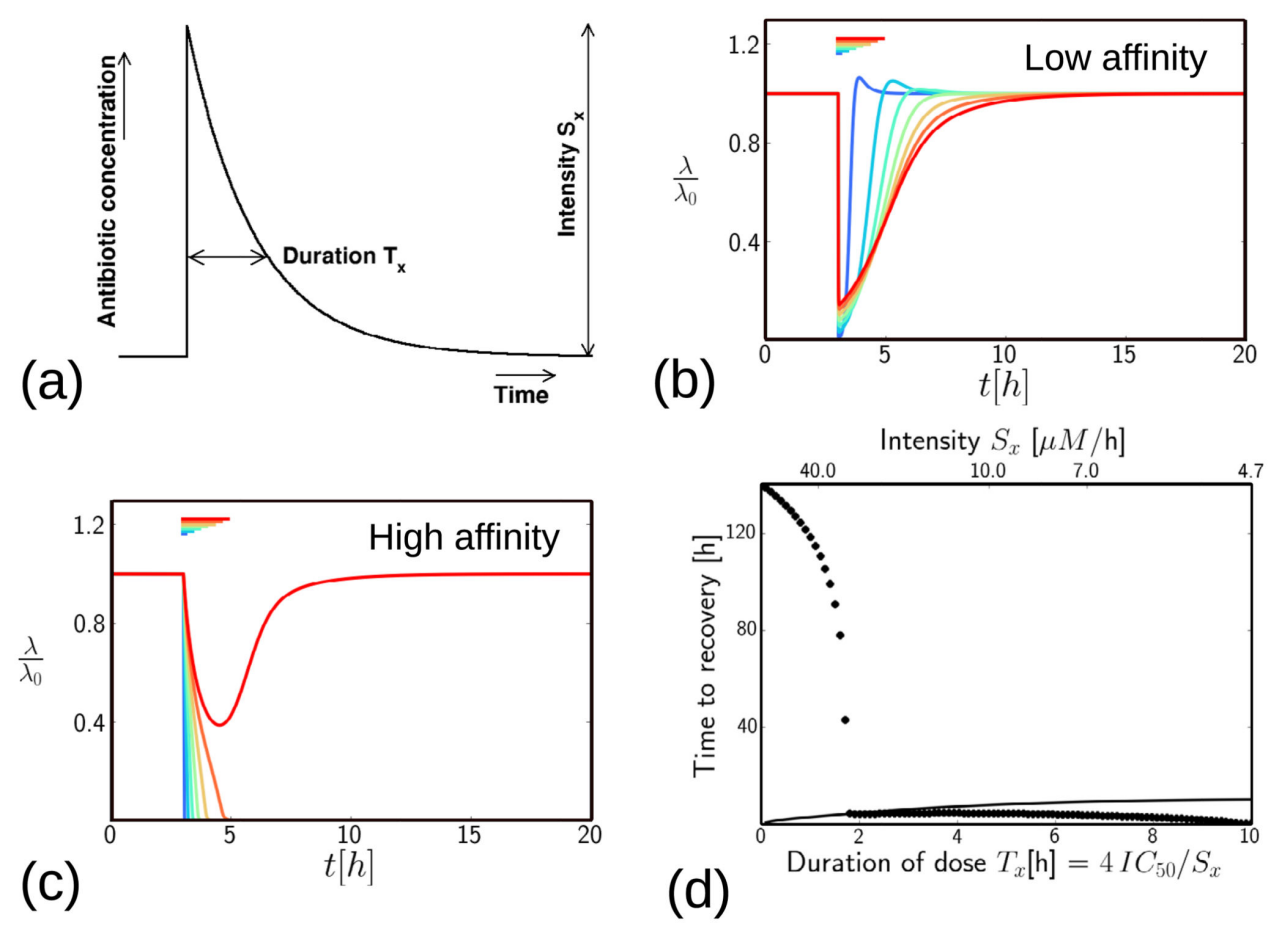
Growth inhibition in response to an exponentially-decaying dose of antibiotic. (a): Illustration of the dosing protocol. The total dose *S*_x_*T*_x_ is fixed at 4 × IC_50_. (b) Results for the low-affinity antibiotic, with parameters as in table 1. The coloured lines represent doses of different duration and intensity, from *T*_x_ = 0.1h (blue) to *T*_x_ = 2.2 h (red), keeping the total dose fixed. The colour bars show the dose duration *T*_x_. (c) Results for the high-affinity antibiotic, with parameters as in table 1. Colours are as in (b). (d) Time taken to recover from a step dose of antibiotic, as a function of the duration / intensity of the dose. ‘Recovery’ is defined as an increase in λ(*t*) to a value 0.9λ_0_, having previously been below this threshold. The recovery time is defined as the total time during which the growth rate is suppressed below 0.9 × λ_0_. The solid line shows results for the low-affinity antibiotic, the symbols show results for the high-affinity antibiotic.

In a clinical scenario, the antibiotic concentration in the body decreases gradually after a dose, rather than suddenly. The pulse profile shown in figure 8(a), in which the concentration increases very rapidly, but decreases exponentially with a decay time *T*_x_, could mimic a dose that is given intravenously and removed by metabolism/excretion. We therefore simulated the response of our model to such a pulse, represented by the function *a*_ex_(*t*) = *S*_x_ exp [−(*t* − *t*_0_)/*T*_x_] for *t* > *t*_0_ = 3 h, and *a*_ex_ = 0 otherwise. We varied the duration *T*_x_, keeping the integrated dose constant: *S*_x_*T*_x_ = 4 × IC_50_.

Figure 8(b) shows results for the low-affinity antibiotic. As for the step pulse, the bacterial growth rate is suppressed during the pulse, to an extent that depends on pulse intensity (the longer, less intense pulse shown by the red curve produces longer duration but weaker growth suppression than the shorter, more intense pulse shown by the blue curve). The model also predicts the same growth-rate overshoot phenomenon for the exponentially decaying pulse which we observed for the step pulse. However, the growth-rate overshoot only happens if the pulse decays quickly enough; for slowly-decaying pulses (large *T*_x_), the overshoot is masked by the growth-rate suppression due to the antibiotic.

The response to an exponentially-decaying pulse of a high-affinity antibiotic (figure 8(c)) is also qualitatively similar to that for the step pulse (figure 7(d)). As for the step pulse, for pulses of intensity below a threshold value, the growth rate recovers quickly following the antibiotic dose. However for pulses with intensity above the threshold, there is a post-antibiotic effect, in which growth suppression persists for long times after the antibiotic has been removed (longer than those shown in figure 8(c)).

Figure 8(d) shows the predicted recovery time after an exponentially-decaying pulse of antibiotic, defined as the time to recover to λ = 0.9λ_0_. For the low-affinity antibiotic (solid line in figure 8(d)), the time to recovery increases with the dose duration. This supports our prediction that for the low-affinity antibiotic, dose duration is the key determinant of treatment efficacy. For the high-affinity antibiotic (symbols in figure 8(d)), the time to recovery shows qualitatively similar behaviour to that for the step-like pulse (compare to figure 7(f)), in that the time to recovery is very long for short, intense pulses, but decreases dramatically for pulses with intensity below a threshold.

We have also performed equivalent simulations for a Gaussian pulse profile, with qualitatively similar results (see appendix C).

Taken together, these results show that the phenomena predicted by our model: (i) duration-dependent efficacy for ribosome-targeting antibiotics which bind with low affinity and/or are transported reversibly, (ii) possible growth-rate overshoot for these ‘low-affinity’ antibiotics, (iii) peak intensity-dependent efficacy for ribosome-targeting antibiotics that bind with high affinity and/or are transported irreversibly and (iv) post-antibiotic effect for these ‘high-affinity’ antibiotics, are all independent of the details of the antibiotic dosage protocol.

## Discussion

In this paper, we have studied the dynamical response of bacterial growth rate to sustained and transient antibiotic treatment, for ribosome-targeting antibiotics. The model that we have used is simple: it includes only antibiotic-ribosome binding, antibiotic transport, growth, and ribosome synthesis, with the latter two processes being dependent on the state of the system. In previous work [8], this model has been shown to predict qualitatively different steady-state behaviour for two classes of ribosome-targeting antibiotics: ‘low-affinity’ antibiotics which bind to ribosomes with low affinity and/or are transported reversibly across the cell boundary, and ‘high-affinity’ antibiotics which bind with high affinity and/or are transported irreversibly.

Here, we go beyond the steady-state analysis of [8], to investigate the response of the model to dynamical changes in antibiotic concentration. Our results show that low-affinity and high-affinity ribosome-targeting antibiotics show qualitatively different dynamical responses to antibiotic treatment. Low-affinity antibiotics show a non-monotonic response, with a rapid decrease in growth rate upon exposure to antibiotic, followed by a slower partial recovery mediated by up-regulation of ribosome synthesis. Up-regulation of ribosome synthesis during exposure also means that these antibiotics may show a growth rate overshoot upon removal of the antibiotic. In contrast, high-affinity antibiotics show a concentration-dependent response: upon antibiotic exposure, the growth rate decreases very little if the antibiotic concentration is below a threshold given by the bifurcation point of the model dynamics, but it decreases almost to zero upon exposure to antibiotic concentrations above the threshold. Close to the threshold concentration the time taken to reach this maximal inhibition can, however, be very long: this behaviour can be understood by the fact that the dynamical trajectories of the model slow down as they pass close to the location where the two fixed points have merged. Furthermore, the model predicts a pronounced post-antibiotic suppression of growth upon removal of a high-affinity antibiotic, for concentrations above the threshold—a phenomenon that results from hysteresis in the model dynamics.

Mathematical models that integrate the molecular mechanism of antibiotic action with bacterial physiology are rare, and those that do exist mostly consider only the response to a time-invariant antibiotic concentration [2, 7–10]. Of those that do consider time-dependent doses of antibiotic, probably the recent work of Abel zur Wiesch *et al* [11, 12] is closest to ours. In that study, a genetic model for antibiotic transport and target binding is considered, and shown to reproduce a range of pharmacodynamic phenomena. However, target-specific physiology (here, the interplay between ribosome concentration and growth rate) is not considered. Here we show that this interplay can play a key role, leading to qualitatively new features such as growth-medium dependent responses and growth-rate overshoots.

Are the predictions of our model realistic? Of course many factors have not been included in the model. For example, we have assumed throughout that growth rate is determined solely by the active ribosome abundance, via equation (4). Although this relation is well-established for steady-state growth, other factors may come into play during transient growth-rate change. In particular, the growth rate may become limited by the supply of amino acids rather than by the abundance of free ribosomes. This might tend to suppress the growth-rate overshoot predicted by our model for the low-affinity antibiotics. More specifically, during an antibiotic pulse, when translation is inhibited, the total ribosome abundance is close to maximal (*r*_tot_ ≈ *r*_max_ in equation (5)). According to the proteome partitioning model, this increased production of ribosomes comes at the expense of producing metabolic enzymes necessary for amino acid supply [20, 26]. Thus, when the antibiotic is removed and ribosomes are released, there may be a transient period when the rate of growth is limited by amino acid supply, before metabolic enzymes are resynthesized to restore the balance between amino acid influx and the demands of translating ribosomes [26]. In this scenario, we would still expect an overshoot in the total ribosome concentration upon removal of the antibiotic, but this might not be coupled to an increase in growth rate. Our model also neglects any other effects of the antibiotics on bacterial physiology: for example, aminoglycosides are believed to increase membrane permeability through the production of misfolded protein [35]. In addition, we do not model bacterial killing, either directly by antibiotic action, or indirectly via the body’s immune system [31]. Inclusion of these killing effects in the model would be likely to prevent the long-time recovery dynamics predicted here for the high-affinity antibiotics.

To conclusively assess the realism of the predictions reported here, one would need experimental tests. Several recently-developed bacterial growth techniques make such tests feasible. At the level of bulk cultures, continuous culture devices have been developed that allow measurement of growth rate during time-dependent antibiotic exposure [28, 36]. Interestingly, turbidostat data for *Enterococcus faecalis* populations exposed to a sudden influx of the ribosome-targeting antibiotic tigecycline, which is expected to be in the low-affinity class, does show rapid growth rate suppression followed by slower partial recovery, as predicted by our model (see figure 1C of [29])^13^. At the level of individual cells, microfluidic devices in which the antibiotic concentration can be changed rapidly as growth is monitored in a microscope are also now possible [30]. The latter would be an especially interesting approach since the bistability which is manifested in our model for high-affinity antibiotics might lead to heterogeneous responses to antibiotic exposure among cells in a population.

If confirmed experimentally, the phenomena reported here would be of considerable clinical significance.In particular, our results make a clear prediction for the optimal pharmacodynamic strategy: for low-affinity drugs one should aim to maximise the time of exposure, while for high-affinity drugs, one should aim to maximise the peak dosage. Moreover, the latter are predicted to show a pronounced post-antibiotic effect, meaning that they can be effective for much longer than the actual duration of exposure. Post-antibiotic effects are a widely recognised, but poorly understood, pharmacodynamic phenomenon, and occur for various antibiotics including aminoglycosides [17–19]. Our work suggests that models that integrate molecular mechanism with bacterial cell physiology can be a useful tool for understanding such clinically relevant growth inhibition phenomena and thus, potentially, for helping to improve clinical practice.

## Supplementary Material

Appendix

## Figures and Tables

**Table 1:** Parameter values used in this study to model low and high-affinity ribosome-targeting antibiotics. These values are chosen to be within the range of the literature values collated in [8]. The universal parameters are *κ_t_* = 6.1 × 10^−2^ *µ*M h^−1^, *r*_min_ = 19.3 *µ*M and *r*_max_ = 65.8 *µ*M [8, 20]. Except where stated otherwise, we have assumed an antibiotic-free growth rate λ_0_ of 1 h^−1^.

Parameter	Value for low-affinity antibiotic	Value for high-affinity antibiotic
*P*_in_	2000 h^−1^	1 h^−1^
*P*_out_	100 h^−1^	0.01 h^−1^
*k*_on_	1000 *μ*M^−1^ h^−1^	1000 *μ*M^−1^ h^−1^
*k*_off_	10^5^ h^−1^	10 h^−1^

$\lambda_{0}^{*}=2\sqrt{P_{\text{out}}\kappa_{t}k_{\text{off}}/k_{\text{on}}}$	49.4 h^−1^	0.00493 h^−1^
$\text{I}{\text{C}}_{50}^{*}=\lambda_{0}^{*}(r_{\max}-r_{\min})/(2P_{\text{in}})$	0.574 *μ*M	0.115 *μ*M
Predicted IC_50_ (from equation (8))	1.43 *μ*M	11.64 *μ*M

## Footnotes

6 The conversion between the units of fraction of cell mass used in [20] and ribosome concentration is discussed in the supplementary material of [8].

7 The assumption that inactive ribosomes do not bind antibiotic simplifies the mathematical analysis; numerical investigations confirm that the qualitative behaviour of the model is the same if the inactive ribosomes are allowed to bind to antibiotic.

8 It is useful to comment on two points regarding the biological interpretation of this model. First, over times shorter than the bacterial generation time, the model describes an exponentially growing bacterial cell. To see this, we note that if the cell is growing exponentially, then its volume increases as $\dot{V}=\lambda V.$ Assuming that molecules of a particular type are produced at a rate proportional to the volume, we obtain *Ṅ* = *gV* for the molecule number *N*, where *g* is a production rate constant. The dynamics of the molecular concentration *n* = *N/V* is then given by $\dot{n}=(1/V)\dot{N}-(N/V^{2})\dot{V}=g-\lambda n,$ as in equations (1)–(3). Second, over times longer than the bacterial generation time, the model describes the behaviour of a lineage of cells. Bacterial cells undergo periodic division events; however, these events do not (on average) change the molecular concentrations, because both the molecule number and the cell volume are (on average) halved. Thus our model effectively describes an experiment in which we follow the dynamics of the molecular concentrations within an individual bacterial cell as it grows and divides, and in which, at each division event, we follow only one of the daughter cells.

9 For details of this calculation see the supplementary material of [8].

10 It is important to recognise that streptomycin and kanamycin have other physiological effects, not included in our model, such as production of misfolded protein which may affect membrane permeability [35].

11 All numerical solutions of the system of ordinary differential equations described in this paper were carried out in python using www.scipy.integrate.odeint.

12 Literature values for kinetic parameters for tetracycline, chloramphenicol, streptomycin and kanamycin are reviewed in the supplementary material of [8].

13 The antibiotic used in figure 1C of [29] is tigecycline (Kevin Wood, personal communication).
