# Supplementary material for: Predicting the dynamics of bacterial growth inhibition by ribosome-targeting antibiotics
Source: Phys Biol. Author manuscript; Available in PMC 2017 Dec 14. (PMC5730049; doi:10.1088/1478-3975/aa8001)
Supplement: Appendix [file NIHMS75308-supplement-Appendix.pdf]

Appendix A. Bifurcation points of the model

As we show in figure 2, the model described by equations (1)–(3) may have different numbers of stationary points (stable and unstable fixed points), depending on the parameter values. Changes in the number and character of the fixed points of the model occur at critical parameter values, and are known as bifurcation points. Figure A1 illustrates these bifurcation points in more detail. Here we use a parameter set intermediate between the low and high-affinity cases studied in the rest of the paper: $P_{\text{in}} = 1\ \text{h}^{-1}$, $P_{\text{out}} = 1\ \text{h}^{-1}$, $k_{\text{on}} = 1000\ \mu\text{M}^{-1}\ \text{h}^{-1}$. We vary the parameter k_{off} and plot the fixed points of the model as a function of $a_{\text{ex}}P_{\text{in}}$. Figure A1(a) shows results with $k_{\text{off}} = 1000\ \text{h}^{-1}$, for which there is only a single fixed point. In contrast, for a smaller value of $k_{\text{off}} = 100\ \text{h}^{-1}$, as shown in figure A1(b), for some values of $a_{\text{ex}}P_{\text{in}}$ the model has one fixed point (which is stable), and for other values of $a_{\text{ex}}P_{\text{in}}$ there are three fixed points, two of which are stable and one unstable. The regime in which there are three fixed points is bistable (shaded) and is bounded by two bifurcation points, labelled $a^*_{\text{ex,low}}$ and $a^*_{\text{ex,high}}$ in figure A1(b), where the number of fixed points changes. The upper bifurcation point $a^*_{\text{ex,high}}$ is associated with a steep decrease in the growth rate λ, since at this bifurcation point the upper stable fixed point is lost and λ drops to the lower fixed point. This critical value $a^*_{\text{ex,high}}$ is very close to (though not exactly equal to) the IC_{50}.

The bifurcation points can be calculated by noting that the fixed points of the model dynamics are given by the roots of equation (7). The number of roots — and thus the number of fixed points—is determined by the discriminant of equation (7): if the discriminant is positive there are three roots, otherwise, there is only one root. Thus the zeros of the discriminant mark the bifurcation points. Since equation (7) is a cubic equation in λ, it may be written as $a\lambda^3 + b\lambda^2 + c\lambda + d$, with discriminant $\Delta = b^2 - 4ac^3 - 4b^3d - 27a^2d^2 + 18abcd$. The zeros of the discriminant can be computed numerically. Figure A1(c) shows the results of such a computation: here the bifurcation points $a^*_{\text{ex,low}}$ and $a^*_{\text{ex,high}}$ are plotted as a function of k_{off}. Since the discriminant itself is cubic in a_{ex} it may have either one or three zeros; those at positive a_{ex} correspond to $a^*_{\text{ex,low}}$ and $a^*_{\text{ex,high}}$[14]. For low values of k_{off} there are two bifurcation points as in figure A1(b), while for high values of k_{off} there is no bifurcation, as in figure A1(a).

We can also obtain an analytical estimate for the upper bifurcation point $a_{\text{ex}} = a^*_{\text{ex,high}}$, which corresponds to the antibiotic concentration at which the model predicts a threshold drop in growth rate. To this end, we rewrite equation (7) in the form

$$\begin{aligned} 0 = \frac{\lambda}{k_{\text{off}}P_{\text{out}}}\left(\frac{a_{\text{ex}}P_{\text{in}}}{\Delta r} + \lambda - \frac{1}{\lambda_0}\lambda^2\right) \\ + \left(\frac{\kappa_t}{k_{\text{on}}}\right)\left[1 + \left(\frac{1}{P_{\text{out}}} + \frac{1}{k_{\text{off}}} - \frac{1}{\lambda_0}\right)\lambda \right. \\ - \left(\frac{1}{P_{\text{out}}\lambda_0} + \frac{1}{k_{\text{off}}\lambda_0} - \frac{1}{P_{\text{out}}k_{\text{off}}}\right)\lambda^2 \\ \left. - \frac{1}{P_{\text{out}}k_{\text{off}}\lambda_0}\lambda^3\right], \end{aligned} \qquad \text{(A.1)}$$

[14] There is also one unphysical zero point of the discriminant at negative values of a_{ex} not shown here.

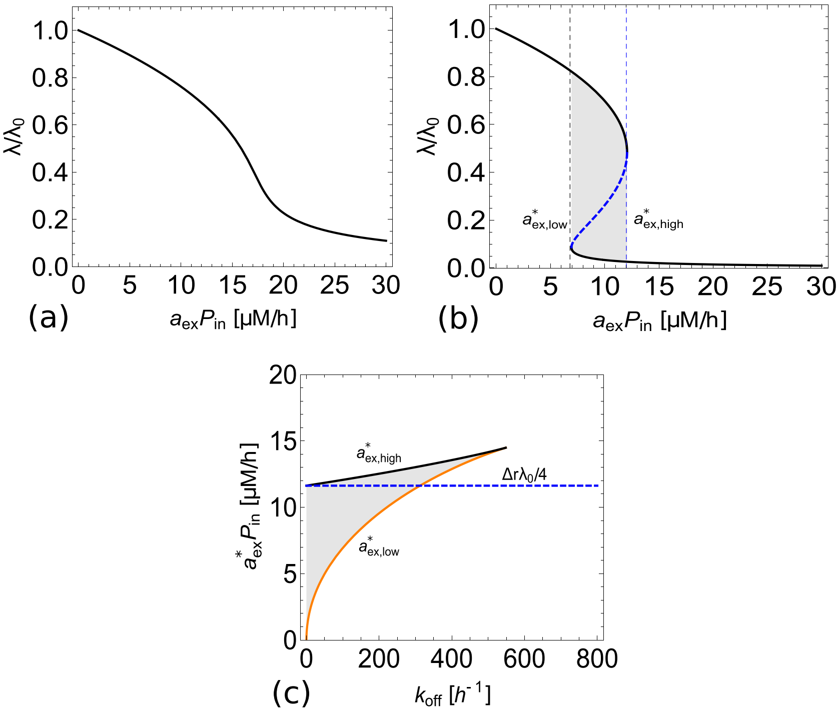

Figure A1. Illustration of the bifurcation points of the model. (a) Growth rate as a function of $a_{\mathrm{ex}}P_{\mathrm{in}}$ for $k_{\mathrm{off}} = 1000\ \mathrm{h}^{-1}$, $P_{\mathrm{in}} = 1\mathrm{h}^{-1}$, $P_{\mathrm{out}} = 1\ \mathrm{h}^{-1}$ and $k_{\mathrm{on}} = 1000\ \mu\mathrm{M}^{-1}\mathrm{h}^{-1}$. For this parameter set the system has one fixed point. (b) Growth rate as a function of $a_{\mathrm{ex}}P_{\mathrm{in}}$ for $k_{\mathrm{off}} = 100\ \mathrm{h}^{-1}$, $P_{\mathrm{in}} = 1\mathrm{h}^{-1}$, $P_{\mathrm{out}} = 1\ \mathrm{h}^{-1}$ and $k_{\mathrm{on}} = 1000\ \mu\mathrm{M}^{-1}\mathrm{h}^{-1}$. Here, the system has regimes with one fixed point and a bistable regime with three fixed points (shaded area)—two of which are stable (solid lines) and one unstable (dashed blue line). The bistable regime is bounded by the critical values $a^*_{\mathrm{ex,low}}$ and $a^*_{\mathrm{ex,high}}$. (c) The critical points $a^*_{\mathrm{ex,low}}$ and $a^*_{\mathrm{ex,high}}$, computed from the discriminant of equation (7) as detailed in the text and plotted as function of k_{off}. The bistable regime is shaded. The blue dashed line marks the analytical estimate $\Delta r\lambda_0/4$ for the upper critical point $a^*_{\mathrm{ex,high}}$, which is valid for $k_{\mathrm{on}} \gg \kappa_t$ and $P_{\mathrm{out}}k_{\mathrm{off}} \lesssim a_{\mathrm{ex}}P_{\mathrm{in}}\lambda/\Delta r$. Note that since equation (7) is symmetric under exchange of k_{off} and P_{out}, $a^*_{\mathrm{ex}}P_{\mathrm{in}}$ as a function of P_{out} would look exactly the same for $k_{\mathrm{off}} = 1\ \mathrm{h}^{-1}$.

(note that we have multiplied equation (7) by a factor of 4). Interestingly, this equation depends only on the combination $a_{\mathrm{ex}}P_{\mathrm{in}}$ rather than on a_{ex} and P_{in} independently. This is why we have used the parameter combination $a_{\mathrm{ex}}P_{\mathrm{in}}$ in figure A1; it also implies that the critical values a^*_{ex} scale as $a^*_{\mathrm{ex}} \sim 1/P_{\mathrm{in}}$. For $k_{\mathrm{on}} \gg \kappa_t$, and $k_{\mathrm{off}}P_{\mathrm{out}}$ not too large, the second term in equation (A.1) can be neglected and we arrive at the quadratic equation

$$0 = \frac{a_{\mathrm{ex}}P_{\mathrm{in}}}{\Delta r} + \lambda + \frac{1}{\lambda_0}\lambda^2. \tag{A.2}$$

The zero of the discriminant is then at

$$a^*_{\mathrm{ex}}P_{\mathrm{in}} = \frac{\Delta r\lambda_0}{4}. \tag{A.3}$$

Setting $r_{\mathrm{min}} = 19.3\ \mu\mathrm{M}$, $r_{\mathrm{max}} = 65.8\ \mu\mathrm{M}$, and $\lambda_0 = 1\ \mathrm{h}^{-1}$, as in table 1, this gives $a^*_{\mathrm{ex}}P_{\mathrm{in}} = 11.625\ \mu\mathrm{M}\ \mathrm{h}^{-1}$, shown in figure A1(c) as the blue dashed line. Figure A1(c) shows that for small values of k_{off} this provides a very good estimate for the upper bifurcation point $a^*_{\mathrm{ex,high}}$. Thus, $a^*_{\mathrm{ex}} = 11.62\ \mu\mathrm{M}\ \mathrm{h}^{-1}/P_{\mathrm{in}}$ is a good estimate for the threshold antibiotic concentration and the IC_{50} for high-affinity antibiotics. Remarkably, this approximation does not explicitly depend on P_{out}, k_{off} or k_{on}. For large values of k_{off} or P_{out}, however, this approximation does not hold anymore, since the prefactor $1/(k_{\mathrm{off}}P_{\mathrm{out}})$ in equation (A.1) decreases the importance of the first term relative to the second term.

Appendix B. Analytical calculation of inhibition time for a high-affinity antibiotic using the adiabatic approximation

Incorporating expression (6) for the ribosome synthesis rate into the dynamical equations (1)–(3), our model can be expressed as:

$$\dot{a} = -\lambda a - k_{\mathrm{on}}(r_{\mathrm{u}} - r_{\mathrm{min}})a + P_{\mathrm{in}}a_{\mathrm{ex}} - P_{\mathrm{out}}a + k_{\mathrm{off}}r_{\mathrm{b}} \tag{B.1}$$

$$\dot{r}_{\mathrm{u}} = -\lambda r_{\mathrm{u}} - k_{\mathrm{on}}(r_{\mathrm{u}} - r_{\mathrm{min}})a + \lambda(r_{\mathrm{max}} - \lambda c) + k_{\mathrm{off}}r_{\mathrm{b}} \tag{B.2}$$

$$\dot{r}_{\mathrm{b}} = -\lambda r_{\mathrm{b}} + k_{\mathrm{on}}(r_{\mathrm{u}} - r_{\mathrm{min}})a - k_{\mathrm{off}}r_{\mathrm{b}} \tag{B.3}$$

where $\Delta = r_{\mathrm{max}} - r_{\mathrm{min}}$, $c \equiv \Delta r(\frac{1}{\lambda_0} - \frac{1}{\kappa_t\Delta r})$ and λ is a function of r_{u} via equation (4). Making the adiabatic approximation, i.e. setting $\dot{a} = 0$, and using equation (4) to eliminate r_{u}, equation (B.1) gives

$$a = \frac{P_{\mathrm{in}}a_{\mathrm{ex}} + k_{\mathrm{off}}r_{\mathrm{b}}}{P_{\mathrm{out}} + \lambda(1 + \frac{k_{\mathrm{on}}}{\kappa_t})}. \tag{B.4}$$

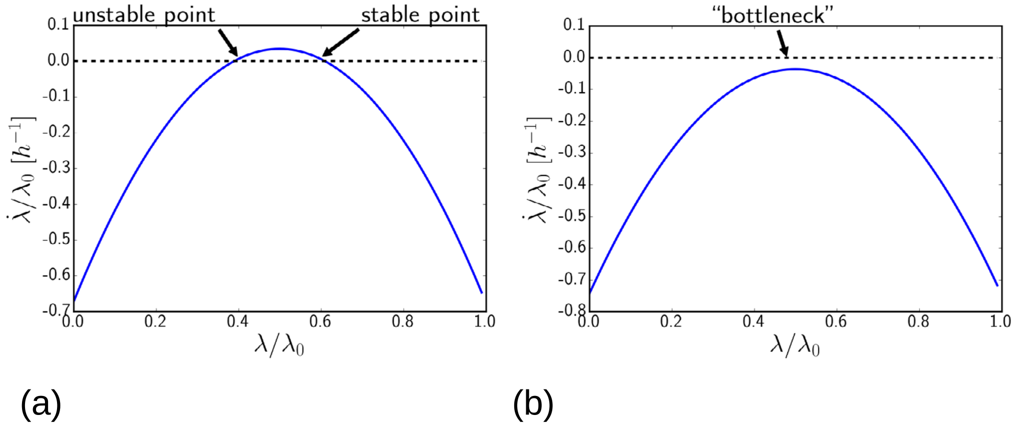

Figure B1. Rate of change of growth rate $\dot{\lambda}$, plotted as a function of λ, as given by the adiabatic approximation, equation (B.7). Panel (a) shows results for $a_{ex} = 0.95 \times IC_{50}$ (just below the bifurcation point of figure 2(b)), while panel (b) shows results for $a_{ex} = 1.05 \times IC_{50}$ (just above the bifurcation point).

Substituting equation (B.4) into equations (B.2) and (B.3) and using equation (4) to change variables from r_u to λ, we obtain

$$\dot{\lambda} = -\lambda^2(1 + c\kappa_t) - \frac{k_{on}\lambda(P_{in}a_{ex} + k_{off}r_b)}{P_{out} + \lambda\left(1 + \frac{k_{on}}{\kappa_t}\right)} + k_{off}\kappa_t r_b + \Delta r\lambda\kappa_t \tag{B.5}$$

and

$$\dot{r}_b = -\lambda r_b + k_{on}\lambda/\kappa_t \frac{P_{in}a_{ex} + k_{off}r_b}{P_{out} + \lambda(1 + \frac{k_{on}}{\kappa_t})} - k_{off}r_b. \tag{B.6}$$

Starting from equation (B.5), we then make the approximation that $\lambda(1 + k_{on}/\kappa_t) \gg P_{out}$[15]. Since we are dealing with a high-affinity antibiotic, we also set $k_{off} = 0$. This allows us to express the model as an equation in one variable only (the growth rate $\lambda(t)$):

$$\dot{\lambda} = -\lambda^2(1 + c\kappa_t) - \frac{k_{on}P_{in}a_{ex}}{(1 + \frac{k_{on}}{\kappa_t})} + \Delta r\lambda\kappa_t. \tag{B.7}$$

Returning to equation (B.7), we can integrate the trajectory $\lambda(t)$ to predict the time T_c required for λ to reach a predefined threshold λ_c:

$$T_c = \int_{\lambda=\lambda_0}^{\lambda=\lambda_c} \frac{d\lambda}{-\lambda^2(1 + c\kappa_t) - \frac{k_{on}P_{in}a_{ex}}{(1 + \frac{k_{on}}{\kappa_t})} + \Delta r\lambda\kappa_t}. \tag{B.8}$$

This integral can be solved using the substitution $u = \Delta r\kappa_t/2 - (1 + c\kappa_t)\lambda\Big/\sqrt{(1 + c\kappa_t)C - (\Delta r\kappa_t)^2/4}$, where we have defined $C = k_{on}P_{in}a_{ex}\Big/1 + \frac{k_{on}}{\kappa_t}$. This gives the following result:

$$\begin{aligned} T_c &= \frac{1}{\sqrt{(1 + c\kappa_t)C - (\Delta r\kappa_t)^2/4}} \int_{u(\lambda_0)}^{u(\lambda_c)} du \frac{1}{1 + u^2} \\ &= \frac{1}{\sqrt{(1 + c\kappa_t)C - (\Delta r\kappa_t)^2/4}} \\ &\times \left(\arctan\left(\frac{\kappa_t\Delta r/2 - (1 + c\kappa_t)\lambda_c}{\sqrt{(1 + c\kappa_t)C - (\Delta r\kappa_t)^2/4}}\right)\right. \\ &\left. - \arctan\left(\frac{\kappa_t\Delta r/2 - (1 + c\kappa_t)\lambda_0}{\sqrt{(1 + c\kappa_t)C - (\Delta r\kappa_t)^2/4}}\right)\right) \end{aligned} \tag{B.9}$$

Setting $\lambda_c = 0.01\lambda_0$ in equation (B.9) leads to the results shown as the solid curve in figure 6. Note that this integration is only valid if $(\Delta r\kappa_t)^2 - 4(1 + c\kappa_t)C < 0$. Otherwise, the denominator becomes zero and T_c diverges. This would be the case if the system is at the upper stable fixed point of the dynamics, such that the growth rate is not significantly decreased upon exposure to the antibiotic.

This analysis also allows us to understand the origin of the very slow inhibition dynamics for the high-affinity antibiotic, for values of a_{ex} just above the bifurcation point, as shown in figure 4(a). Figure B1 shows the rate of change of the growth rate, $\dot{\lambda}$, plotted as a function of λ, as predicted by equation (B.7), for the high-affinity parameter set. Figure B1(a) shows results for $a_{ex} = 0.95 \times IC_{50}$ (just below the bifurcation point): the fixed points correspond to zeroes of $\dot{\lambda}$ and the stable one is indicated by the arrow (there is of course also another stable fixed point at very small λ, but this is lost in the quadratic approximation of equation (B.7)). Figure B1(b) shows equivalent results for a slightly higher antibiotic concentration, $a_{ex} = 1.05 \times IC_{50}$, just above the bifurcation point. Here the fixed points are lost, but the rate of change of λ still comes close to zero, implying that the speed of inhibition by the anti-

[15] We expect this approximation to be valid close to the upper fixed points, whose bifurcation we are concerned with here. Close to the lower stable fixed point, where $\lambda \to 0$, the approximation may not hold.

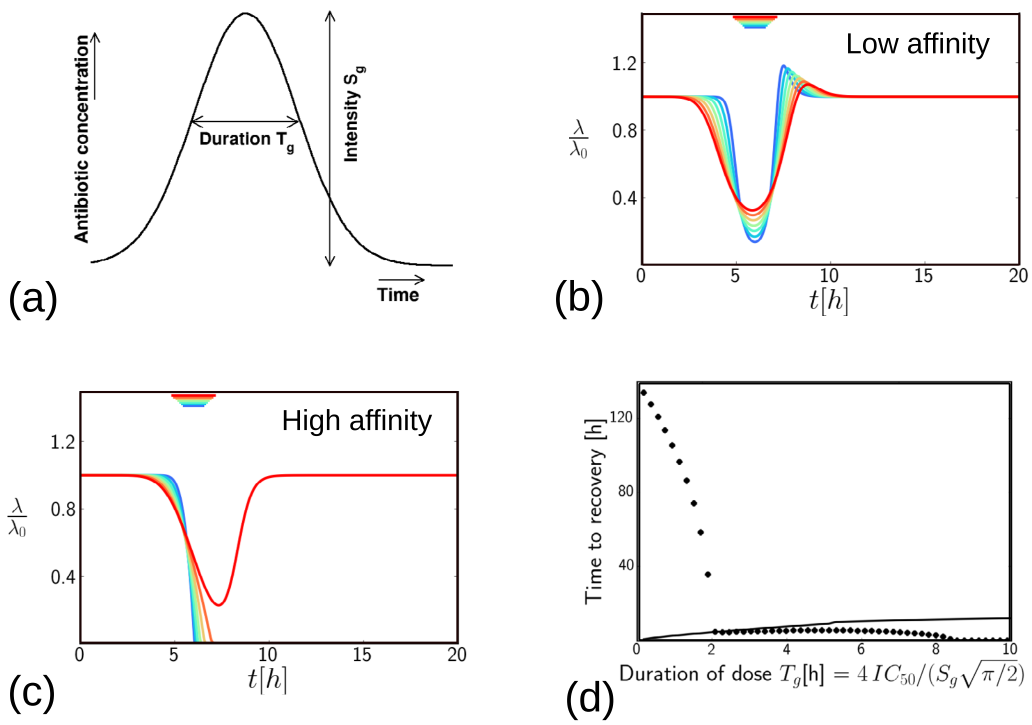

Figure C1. Growth inhibition in response to a transient dose of antibiotic with a Gaussian profile. (a): Illustration of the dosing protocol. The total dose, which is given by $\sqrt{(\pi/2)}\,S_g T_g$, is fixed at $4 \times IC_{50}$ and the time of the peak dose $t_{max} = 6$ h. (b) Results for the low-affinity antibiotic, with parameters as in table 1. The coloured lines represent doses of different duration and intensity, from $T_g = 0.8$ h (blue) to $T_g = 2.2$ h (red), keeping the total dose fixed). The colour bars show the duration T_g of the dose. (c) Results for the high-affinity antibiotic, with parameters as in table 1. Colours are as in (b). For all curves except the red one, the growth rate eventually recovers to its pre-dose value, on a timescale that is longer than shown in the plot. (d) Time taken to recover from a step dose of antibiotic, as a function of the duration of the dose. 'Recovery' is defined as an increase in $\lambda(t)$ to a value $0.9\lambda_0$, having previously been below this threshold. The recovery time is defined as the total time during which the growth rate is suppressed below $0.9 \times \lambda_0$. The solid line shows results for the low-affinity antibiotic, the symbols show results for the high-affinity antibiotic.

biotic will be very slow. This slow dynamics close to the bifurcation point can be thought of as a 'bottleneck' in the inhibition trajectory.

Appendix C. Response to a Gaussian pulse of antibiotic

As an example of a dosage profile without any discontinuities, we also simulated the response of the model to a Gaussian pulse of antibiotic, of the form $a_{ex}(t) = S_g \exp[-2(t - t_{max})^2/T_g^2]$, as shown in figure C1(a). We varied the intensity S_g and the duration T_g, keeping the integrated dose, which is given approximately by $\sqrt{(\pi/2)}\,S_g T_g$, fixed at $4 \times IC_{50}$. The results are qualitatively similar to those for the step-dose and the exponentially decaying dose, described in the main text. For the low-affinity antibiotic, figure C1(b) shows that the bacterial growth rate is suppressed during the Gaussian pulse, to an extent that depends on pulse intensity, and we see the same growth-rate overshoot phenomenon following the Gaussian antibiotic pulse which we observed for the step and exponentially-decaying pulses. For the high-affinity antibiotic, figure C1(c) shows that for pulses of intensity below a threshold value, the growth rate recovers quickly following the antibiotic dose, but for pulses with intensity above the threshold growth suppression persists for long times after the antibiotic has been removed. Figure C1(d) shows the predicted recovery time after a Gaussian pulse of antibiotic, defined as the time to reach $\lambda = 0.9\lambda_0$. For the low-affinity antibiotic (solid line in figure C1(d)), the time to recovery increases with the dose duration, whereas for the high-affinity antibiotic (symbols in figure C1(d)), the time to recovery is very long for short, intense pulses, but decreases dramatically for pulses with intensity below a threshold. The more complex shape of the plot for low-intensity pulses, compared to the results for the step and exponentially-decaying pulses, is due to the shape of the Gaussian pulse; for values of T_g above ~9 h, the growth rate no longer decreases below $0.9\lambda_0$.
